# Recruitment of homodimeric proneural factors by conserved CAT–CAT E-boxes drives major epigenetic reconfiguration in cortical neurogenesis

**DOI:** 10.1093/nar/gkae950

**Published:** 2024-11-04

**Authors:** Xabier de Martin, Baldomero Oliva, Gabriel Santpere

**Affiliations:** Neurogenomics Group, Hospital del Mar Research Institute, Parc de Recerca Biomèdica de Barcelona (PRBB), Dr. Aiguader, 88, Barcelona 08003, Catalonia, Spain; Structural Bioinformatics Lab (GRIB-IMIM), Department of Medicine and Life Sciences, Universitat Pompeu Fabra, Dr. Aiguader, 88, Barcelona 08003 Catalonia, Spain; Neurogenomics Group, Hospital del Mar Research Institute, Parc de Recerca Biomèdica de Barcelona (PRBB), Dr. Aiguader, 88, Barcelona 08003, Catalonia, Spain; Department of Neuroscience, Yale School of Medicine, 333 Cedar st., New Haven, CT 06510, USA

## Abstract

Proneural factors of the basic helix–loop–helix family coordinate neurogenesis and neurodifferentiation. Among them, NEUROG2 and NEUROD2 subsequently act to specify neurons of the glutamatergic lineage. Disruption of these factors, their target genes and binding DNA motifs has been linked to various neuropsychiatric disorders. Proneural factors bind to specific DNA motifs called E-boxes (hexanucleotides of the form CANNTG, composed of two CAN half sites on opposed strands). While corticogenesis heavily relies on E-box activity, the collaboration of proneural factors on different E-box types and their chromatin remodeling mechanisms remain largely unknown. Here, we conducted a comprehensive analysis using chromatin immunoprecipitation followed by sequencing (ChIP-seq) data for NEUROG2 and NEUROD2, along with time-matched single-cell RNA-seq, ATAC-seq and DNA methylation data from the developing mouse cortex. Our findings show that these factors are highly enriched in transiently active genomic regions during intermediate stages of neuronal differentiation. Although they primarily bind CAG-containing E-boxes, their binding in dynamic regions is notably enriched in CAT–CAT E-boxes (i.e. CATATG, denoted as 5′3′ half sites for dimers), which undergo significant DNA demethylation and exhibit the highest levels of evolutionary constraint. Aided by HT-SELEX data reanalysis, structural modeling and DNA footprinting, we propose that these proneural factors exert maximal chromatin remodeling influence during intermediate stages of neurogenesis by binding as homodimers to CAT–CAT motifs. This study provides an in-depth integrative analysis of the dynamic regulation of E-boxes during neuronal development, enhancing our understanding of the mechanisms underlying the binding specificity of critical proneural factors.

## Graphical Abstract

**Graphical Abstract:**
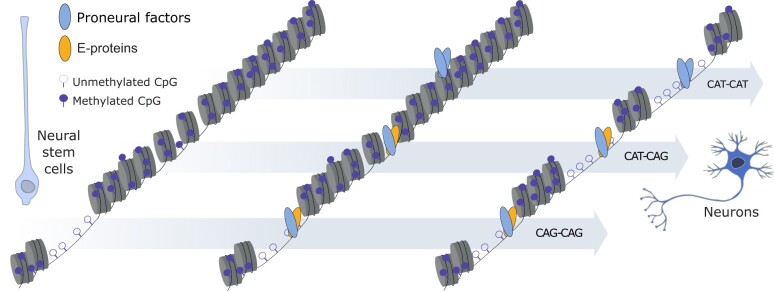

## Introduction

Mammalian cortical neurogenesis initiates when the self-renewing population of neural stem cells (NSCs) begins to divide asymmetrically to give rise to neurons or intermediate progenitor cells (IPCs), which in turn can go through several proliferative divisions before these undergo a final round of terminal division to become postmitotic neurons. Initially, the newborn embryonic neurons undergo morphological changes, transitioning from multipolar to bipolar and migrate towards the cortical plate, along the basal process of the NSCs (1). Finally, the neurons settle in the cortical plate and undergo terminal differentiation, a process that includes sending their axons to distant targets, growing dendrites and forming synapses (2–4). These dynamic processes imply intermingled axes of cellular differentiation and maturation (5), which are accompanied with extensive modifications of the transcriptome and epigenome. These major reconfigurations are primarily orchestrated by a subset of transcription factors (TFs) (6–8).

It is well established that proneural TFs of the basic helix–loop–helix (bHLH) family represent major early coordinators of the gene regulatory networks that drive cortical neurodevelopment (9,10). Within this group, the expression of Neurogenins in neural progenitors is necessary and sufficient to specify a glutamatergic neuronal lineage, while simultaneously ⁠inhibiting the GABAergic lineage (10,11), and they redundantly mediate the acquisition of the laminar identity of deep-layer neurons (12). Neurogenins have been also implicated in neuronal migration, for instance enabling the multipolar–bipolar transition by activating the expression of *Rnd2* (13). Furthermore, Neurogenin 2 (NEUROG2) has been shown to activate NeuroD genes in IPCs and early born neurons (14,15). This subsequent expression of NeuroD factors (*Neurod1, Neurod2 and Neurod6*) mediates the process of maturation of the newborn neurons into fully differentiated excitatory glutamatergic neurons (16)⁠. Indeed, a ChIP-seq analysis of NEUROD2 in the mouse brain followed by validation experiments indicated that NEUROD2 regulates the expression of many key TFs responsible for neuronal subtype specification (e.g. Fezf2, Bcl11b, Satb2 and Cux1) (17), placing NeuroD factors at the root of glutamatergic neuron ontogeny.

Knockout and functional analyses revealed that NeuroD factors jointly regulate several aspects of migration and axonogenesis (18)⁠. Single knockouts reported milder defects in neuronal migration and axonogenesis than double or triple knockouts, supporting their functional redundancy in those early processes (18,19)⁠. The expression of these factors diverges in the mature neurons that have settled in the cortical plate: while the expression of *Neurod2* and *Neurod6* is maintained lifelong, albeit at lower levels, the expression of *Neurod1* is lost. Consistent with this later decoupling, knockout *Neurod2* mice show more severe defects in the functional characteristics of mature neurons, specifically in synaptic functions and excitability, and also present with epilepsy and autistic-like behavior (20,21)⁠. Interestingly, *NEUROD2* and its target genes exhibit enrichment in genetic variants associated with various neuropsychiatric phenotypes, most prominently, autism spectrum disorder (ASD) (21–24).

Proneural factors, like most bHLHs, modulate gene expression by binding to DNA in dimeric form, either as homodimers (25–27), by forming heterodimers with other proneural factors [e.g. NEUROG1–NEUROG2 (25), NEUROG2–ASCL1 (28,29) or NEUROG2–NEUROD4 (30)], or with E-proteins, which are encoded by a more broadly expressed group of bHLH genes: *Tcf4*, *Tcf3* and *Tcf12* (31–33). Notably, mutations in *TCF4* cause Pitt–Hopkins syndrome (32), a neurodevelopmental disorder characterized by intellectual disability and developmental delay and have also been associated with schizophrenia and ASD. In addition, TCF4 targets have also been associated with schizophrenia (34,35). Several studies have shown that experimental perturbation of *Tcf4* disrupts multiple aspects of neurodevelopment, predictably showing phenotypic overlaps with knockouts of Neurogenins and NeuroD factors, including the pace of neurogenesis, neuronal migration, morphology and subtype specification, dendrite and synapse formation and the establishment of interhemispheric connections (35–38).

BHLH TFs bind a consensus CANNTG motif known as the E-box, being the preference for the central dinucleotide variable among the subclasses of bHLH factors. As each bHLH monomer binds one CAN half-site in opposing strands, we can refer to E-boxes by their 5′–3′ oriented half-sites. Heterodimers of proneural factors with E-proteins bind preferentially to CAT–CAG motifs (32,39,40)⁠, and also to CAG–CAG and CAG–CAC motifs (39,41). Importantly, it has been shown that different functional outcomes can arise from variations in E-box usage. For instance, when NEUROD2 binds to DNA via the CAG–CAG motif, these binding sites are more frequently shared with other non-neuronal bHLH factors, are linked to a reduced ability to activate target gene expression, and the associated target genes are involved in general cellular processes (42). Conversely, CAT–CAG motifs are bound more specifically by NEUROD2, the target genes associated to these motifs are more strongly transactivated and are associated with neuronal functions (42). However, the correlation between E-box preference and neuronal developmental processes *in vivo* remains unknown.

In recent years, large-scale single-cell analyses have enabled the evaluation of cell type-specific developmental trajectories of chromatin accessibility in the neocortex. DNA motif enrichment analyses in these dynamic regions showed that E-boxes associated with proneural factors are the most highly enriched motifs in regions that become accessible and peak during early stages of neurogenesis (7,43–46). Furthermore, E-boxes have also been found to be significantly enriched in regions involved in chromatin looping and that undergo CpG demethylation in neurodifferentiation, with NEUROG2 and NEUROD2 proposed as contributors to these effects (47–50). Importantly, numerous E-boxes residing in the accessible chromatin regions of developing neurons span mutations associated with ASD and Parkinson’s disease (44,51)⁠.

While neurogenesis and neurodifferentiation appear to rely heavily on the activity of E-boxes, our understanding of the precise E-box utilization by the proneural bHLH factors and E-proteins remains limited. To shed light on this critical aspect of neuronal development, we conducted a comprehensive analysis leveraging ChIP-seq data of NEUROG2 and NEUROD2, in conjunction with time-matched single-cell gene expression and chromatin accessibility data obtained from the developing mouse cortex. Our analyses reveal that NEUROG2 and NEUROD2 binding sites are highly biased towards genomic regions that become accessible in neurogenesis, and that these binding sites are remarkably enriched in the evolutionarily constrained CAT–CAT E-boxes. Finally, we harmonize observations derived from multiomics, detailed DNA-footprinting, HT-SELEX and structural modeling, into a comprehensive model delineating the combinatorial mechanisms of proneural factors and E-proteins throughout neurogenesis.

## Materials and methods

### ChIP-seq data processing

Raw public NEUROD2 ChIP-seq data of E14.5 NEUROD2, P0 NEUROD2 and E14.5 NEUROG2 were obtained from the Gene Expression Omnibus database, with accession numbers GSE67539, GSE63620 and GSE84895, respectively. FASTQ reads were aligned to the mouse reference genome mm10 using the bwa-mem program of BWA version 0.7.17 (52) with default parameters. Duplicated reads were removed with SAMtools version 1.12. (53). We kept uniquely mapped reads with the following regular expression: ‘grep -v -e “XA:Z:” -e “SA:Z:”’. We called peaks with MACS3 (54) specifying a bandwidth of 200 and using the ChIP-seqs of the GFP of the corresponding replicate as a control, and with default values for all other parameters. We used only replicates 1 and 2 in both E14.5 and P0 samples, since replicate 3 added almost no additional peaks (Supplementary Figure S1B and C). For each replicate, peaks were divided into subpeaks using the PeakSplitter program (55). A final set of consensus peaks was obtained by intersecting subpeaks from the two first replicates of the E14.5 and P0 NEUROD2 experiments (NEUROG2 had only one replicate), with the requisite that the summits of both peaks fall within the intersection. Venn diagrams of the intersection between NEUROG2 and NEUROD2 peaks and among their replicates were plotted using the *eulerr* R package (56).

### Single-cell ATAC-seq data processing

We obtained single-cell ATAC-seq raw FASTQ files of the E14.5 mouse cortex experiment by Noack *et al.* (2023) from SRA (accession: SRP275808). Reads were aligned to the mouse mm10 assembly using the *count* function of Cell Ranger ATAC version 2.1 (57). We took cell annotations imputed from the single-cell mRNA sequencing (scRNA-seq) assay of the same study, generously provided to us by Noack *et al.* We then made subsets of the aligned reads based on authors’ cell-type annotation, taking 350 random cells from each major cell cluster: NSC, IPC, PN1, PN2 and PN3. Bam files from replicates 1 and 2 were merged, and then merged bam files of the selected cell-types were joined into a single bam file representing the excitatory neuron lineage. Peak calling was performed using the MACS2 program (54), and the resulting peaks were subsequently divided into subpeaks with PeakSplitter. Subpeaks were centered around the summit and resized to 500 bp. Next, taking those peaks and the fragments files of the single-cell ATAC-seq assay (GEO accession: GSE155677), we generated count matrices, with the FeatureMatrix function of Signac (58).

To obtain developmental trajectories of chromatin accessibility, we first ordered cells based on their pseudotime values imputed from the scRNA-seq assay and made groups of 175 cells based on that ordering: eight groups for NSCs, five for IPCs, seven for PN1, eight for PN2 and two for PN3. Counts from each group of cells were aggregated into pseudobulk samples and normalized as log_2_ counts per million (CPMs) reads. We tested peaks for significant differences in accessibility across cell clusters by means of an analysis of variance (ANOVA). Peaks with *P*-values ≥ 10^−06^ were classified as invariant. The rest of the peaks were classified as dynamic. In order to group those peaks in developmental trajectories, we first generated binary vectors with each position corresponding to one cell pseudobulk (0s and 1 s, representing closed or accessible chromatin, respectively). That way, we generated combinations of 0s and 1s for predefined developmental trajectories. For example, the ‘000000000000000000000000000011’ vector represents that the peak is closed in NSCs, IPCs, PN1s, PN2s and then opened in PN3s, and the ‘000000001111111111110000000000’ represents peaks that are closed in NSCs, open in IPCs and PN1s and then closed again. Then, we took each one of the peaks, and we performed Pearson correlations between the vector representing its accessibility in the successive cell pseudobulks with each one of the binary vectors representing the different developmental trajectories. The peaks were labeled with the trajectory whose binary vector correlated the most with their accessibility. To sort the ATAC-seq peaks based on the correlation with the expression of *Neurog2* and *Neurod2*, we used the Seurat scATAC-seq object with imputed scRNA-seq expressions, kindly provided to us by Noack *et al.* (50). We sorted cells based on the imputed SCT-normalized expression of the TF of interest, performed pseudobulk groups of 50 cells based on that ordering and correlated the mean sctransform (SCT)-imputed expression of *Neurog2* and *Neurod2* in the pseudobulk samples with the mean accessibility of each peak in those pseudobulk samples.

Open chromatin regions of the P56 mouse cortex were obtained from Li *et al.* (59) online resource: http://catlas.org/catlas_downloads/mousebrain/cCREs/. We merged all peaks called in the each subtype of excitatory neurons into one single set. We obtained raw single-cell ATAC-seq matrix from multiple tissues derived from adult mice (60) from https://atlas.gs.washington.edu/mouse-atac/data/#release-updates. A peak was considered to be accessible in a given cell cluster if it had at least one count in at least 3% of the cells.

### Gene ontology analysis

To assign genes to NEUROD2 peaks, we made use of the ATAC-seq peak-gene links based on the positive correlation between the accessibility of the peaks and the imputed expression of proximal genes, provided in the supplementary materials of Noack *et al.* (50). A gene was considered linked to a NEUROD2 peak if the NEUROD2 peak intersected with an ATAC-seq peak that positively correlated with the gene.

For the gene ontology analysis of the NEUROD2 peaks in the trajectories, odds ratios (ORs) were computed as the fraction of genes in each trajectory associated to each ontology term divided by all the genes in that trajectory versus the number of genes associated to each ontology term divided by all the genes used as background by *g:Profiler* (61).

### E-box enrichment and centrality

For the motif centrality analysis in the ATAC-seq peaks, BED files with the occurrences in the mm10 genome of archetypal motifs derived by Vierstra *et al.* (62) were obtained from https://resources.altius.org/∼jvierstra/projects/motif-clustering-v2.0beta/. To compute the centrality of those archetypal motifs in the ATAC-seq peaks, peaks were resized to 1500 bp around their summits and the occurrences of the motifs were counted in two windows: one 50 bp around the summit of the peaks and the other 250 bp further from the summit. The centrality was computed as the fold change of the occurrences of motifs per base-pair in the window around the summit versus the distal window. As a result, motifs that have a centrality value > 1 are centrally enriched, and the ones that are < 1 are centrally depleted.

### Measurement of CpG methylation levels in E-boxes

BigWig files with whole-genome %CpG methylation for sorted neuronal cells of the embryonic mouse cortex were obtained from an online resource facilitated by Bonev’s lab: https://bonevlab.com/resources. The %CpG methylation data of the excitatory neurons of the adult mouse brain were downloaded as TSV files from http://neomorph.salk.edu/hanqingliu/cemba/ALLC/MajorType/, converted to bedGraph files, and then to BigWig files using the *bedGraphToBigWig* program (63). BigWig files were used as input for the methylation plots. The %CpG methylation heatmaps were plotted using the *EnrichedHeatmap* package (64), averaging %CpG methylation values in windows of 50 bp with the ‘absolute’ method and applying smoothing between the rows of the matrix. The lineplots were produced with the *SeqPlots* package (65), averaging the %CpG methylation in bins of 10 bp.

### Analysis of HT-SELEX data

Raw FASTQ files for each of the four HT-SELEX rounds performed by Jolma *et al.* (66) were downloaded from the European Nucleotide Archive, under accession number ERP001824. For each round, the hexanucleotides were identified in the FASTQ files by means of regular expressions, and the percentage of sequences that carried each hexanucleotide was computed.

### Analysis of TF induction experiments

We gathered data from ATAC-seq, H3K27ac ChIP-seq and H4ac ChIP-seq studies on cells undergoing Neurog2 or Neurod2 induction (42,67–71). Reads were processed as described above for NEUROG2 and NEUROD2 ChIP-seq, using the macs2 program with the broad parameter specified for chromatin modifications. NEUROG2 and NEUROD2 ChIP-seq data from these induction experiments were processed similarly, except for those already reprocessed in the ReMAP2022 database, which employs the same methods.

To classify ChIP-seq peaks based on pre-induction chromatin accessibility, we used .bedGraph files containing reads per kilobase of transcript, per million mapped reads (RPKM)-normalized signals of chromatin accessibility, mapped within a window around the peak summit (2 kb for ATAC-seq and 4 kb for histone modification ChIP-seqs). The mean accessibility was calculated using the bedtools map function from BEDTools, and peaks were subsequently sorted based on this mean. For motif enrichment, the mean number of motifs per peak was calculated in a window proportional to peak size (0.4 of its length).

For studies that measured chromatin accessibility both before and after TF induction (42,68,69), we performed peak calling independently for each condition, merged the resulting peaks into a single set and mapped chromatin accessibility signals before and after induction onto this set. The mean signal was calculated, and peaks were sorted by the fold change in post- versus pre-induction accessibility.

### ModCRE

ModCRE is a structure-based method for predicting TF binding motifs, represented as position weight matrices (PWMs), and automatically modeling the structure of protein–DNA complexes. It relies on statistical potentials derived from both Protein Data Bank (PDB) structures and TF–DNA interactions documented in protein binding microarrays (PBM) experiments (72). Specifically, ES3DCdd statistical potentials, as described in Meseguer *et al.* (73), have been found most suitable for predicting PWMs and assessing a TF’s DNA binding capability. The interaction score for a TF–DNA pair is determined by summing the scores of all contact pairs between amino acids and nucleotides within the interaction. To compare different complexes, we employ -ES3DCdd as a measure of binding affinity, where higher values indicate stronger binding. We assess the interaction strength of a specific nucleotide within the binding site by considering all its contacts with interface amino acids, including cases with two or more proteins in the interaction (as seen in dimers and macro-complexes). We applied ModCRE to model the structures of both heterodimers and homodimers involving NEUROD2, NEUROG2 and TCF4. We predicted the PWMs and evaluated their binding affinities. To account for structural variability and flexibility, we considered multiple conformations and binding preferences generated from various templates. This approach enhances the robustness of comparisons between homodimers and heterodimers in our study. The templates used for this analysis were 2QL2, 2YPA, 2YPB, 6OD3, 6OD4, 6OD5 and 1MDY.

### Analysis of E-box sequence conservation

For the genomic variation analysis, we analyzed a VCF file containing data of a population of 154 wild mice, kindly provided to us by Lawal *et al.* (74). Variants were filtered with GATK (75), using the following expression: ‘QD < 2.0 || FS > 60.0 || MQ < 40.0 || MQRankSum < −12.5 || ReadPosRankSum < −8.0’. Then, we removed the indels with *VCFtools*, and converted the filtered VCF files into BED files. We considered single nucleotide variants (SNVs) as polymorphic if they had a minor allele frequency superior or equal to 0.05. In order to assess the rate of polymorphisms in each E-box type, polymorphic SNVs were intersected with the E-boxes bed file and counted. To study the divergence with respect to the rat genome, the mm10 versus the rn7 genome alignment was downloaded (https://hgdownload.soe.ucsc.edu/goldenPath/mm10/vsRn7/mm10.rn7.synNet.maf.gz), and number of single nucleotide substitutions that occurred within the hexanucleotides were counted, excluding the motifs with indels.

For the evolutionary divergence analysis, we downloaded multiple alignments of the mm10 assembly against 60 species of vertebrates for each chromosome as multiple alignment format (MAF) files from http://hgdownload.soe.ucsc.edu/goldenPath/mm10/multiz60way/maf/. The alignments of our motifs of interest were extracted from those MAF files using the *mafsInRegion* tool provided by UCSC (http://hgdownload.cse.ucsc.edu/admin/exe/linux.x86_64/mafsInRegion), and converted to FASTA files with the maf_to_concat_fasta.py program (https://github.rcac.purdue.edu/kelley/opt-galaxy/blob/master/eggs/bx_python-0.7.2-py2.7-linux-x86_64-ucs4.egg/EGG-INFO/scripts/maf_to_concat_fasta.py). The FASTA files were imported to R using the *read.fasta* function of the *seqinr* package (76) and subsequently analyzed in R-based in-house scripts. For the evolutionary analysis, a motif was considered to be conserved in a given species versus the rat, if it had 0 substitutions in the core CANNTG hexanucleotide. Only single-nucleotide substitutions were considered, and motifs spanning indels were excluded. To assign the phylostratigraphical depths to the motifs, we required the motif to be conserved in a proportion of species of a given phylostratigraphical depth; 20 out of 39 placental mammals, 1 out of 3 marsupials, 1 out 1 monotreme and 8 out of 16 non-mammals.

## Results

### Transcriptional dynamics of proneural factors and E-proteins during mouse neurogenesis

**Figure 1.**
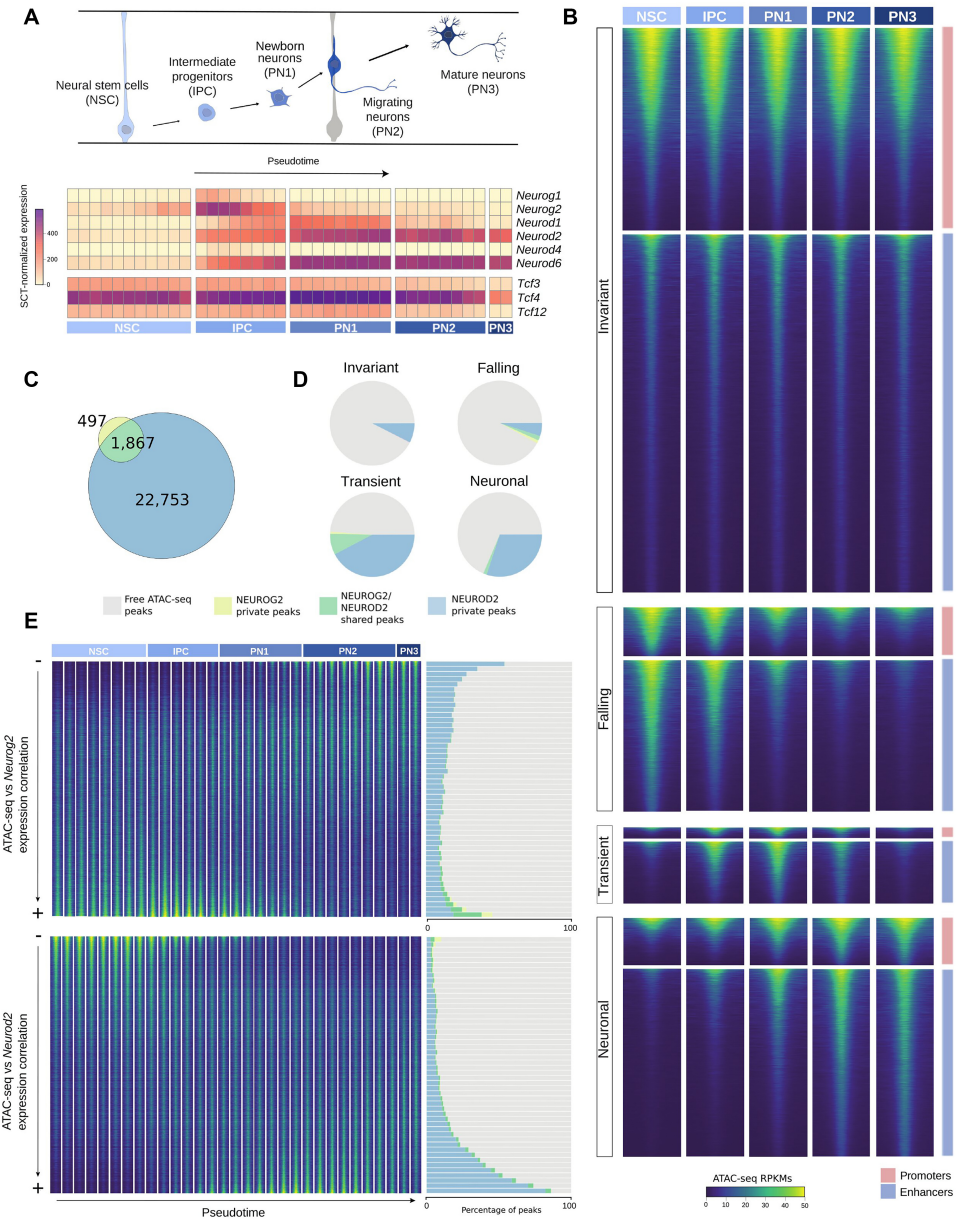
Mapping NEUROG2 and NEUROD2 binding sites to the chromatin accessibility landscape of corticogenesis. (**A**) Simplified model of cortical neurogenesis, depicting the main cell types of the projection neuron lineage (top). Mean expression of proneural factors and E-proteins in bins of cells ordered by pseudotime (bottom). (**B**) Heatmaps depicting ATAC-seq signals measured as RPKMs in each cell-type pseudobulk, where each row represents a region of 1500 bp around the summit of the ATAC-seq peaks. Regions are grouped by their main trajectories, and further subdivided by the localization within promoter or enhancer regions. (**C**) Venn diagram representing the relative amount of private, or enriched and shared binding sites of NEUROG2 and NEUROD2. (**D**) Proportion of ATAC-seq peaks in each trajectory that are bound by private NEUROG2 or NEUROD2 peaks, by shared peaks, or not bound by any of them. (**E**) (Left) Heatmap representing average pseudobulk signal around the summits of the ATAC-seq peaks sorted by the correlation of their accessibility with the expression of the genes. Pseudobulk samples are composed of cells grouped in increasing pseudotime bins. (Right) Proportion of ATAC-seq peaks bound by NEUROG2 and NEUROD2 in 50 groups of equal size, made based on the same ordering in the heatmap Y-axis.

We set up to investigate the mechanisms of DNA binding and action of proneural factors during cortical neurogenesis. After an exhaustive screening of appropriate ChIP-seq datasets (77), we identified and gathered good quality ChIP-seq data for NEUROD2 (17) and NEUROG2 (78) together with single-cell transcriptomics and chromatin accessibility data (50), all derived from mouse cortex at embryonic day 14.5 (E14.5), a period of major neurogenic activity. Prior to the ChIP-seq analysis, we characterized the expression of Neurogenins, NeuroD factors and E-proteins throughout the entire neurogenic axis, encompassing NSCs, intermediate progenitor cells (IPCs) and three populations of excitatory projection neurons: newborn neurons (PN1), migrating neurons (PN2) and mature neurons (PN3) (Figure 1A). These data show that the expression of *Neurog2* begins in NPCs and peaks in IPCs, a pattern that is mirrored by *Neurog1*, albeit with lower expression levels. Conversely, the expression of *Neurod2* and *Neurod6* starts in IPCs but reaches its peak in immature excitatory neurons (PN1 and PN2) and it remains detected in mature neurons. In contrast, *Neurod1* demonstrated later and weaker expression in immature neurons, gradually disappearing at PN2 (Figure 1A). The fluctuating expression of the proneural factors contrasted to that of E-proteins, of which *Tcf4* consistently maintained the highest expression level across the entire developmental axis.

### NEUROG2 and NEUROD2 binding in the chromatin accessibility landscape of mouse cortical neurodevelopment

We next aimed to position the binding of NEUROG2 and NEUROD2 within the chromatin accessibility map of cortical neurogenesis. To achieve this, we first reanalyzed ChIP-seq data for NEUROG2 and NEUROD2 in the E14.5 mouse cortex, identifying 2356 and 26463 peaks, respectively. The intersection of both datasets revealed NEUROG2 or NEUROD2 enriched peaks, as well as shared peaks surpassing the detection threshold for both TFs (see the ‘Materials and methods’ section). NEUROD2 technical replicates demonstrated highly concordant binding profiles (Supplementary Figure S1A and B). This smaller number of NEUROG2 peaks compared to NEUROD2 might be attributed to technical and biological reasons, since at E14.5 the bulk tissue might overrepresent peaks occurring in nascent neurons, where NEUROD2 has maximum expression. Additionally, at E14.5, the neurogenic activity of NEUROG2 has been found to be attenuated compared to that of earlier embryonic stages (12,26). Despite the assumption of technical variation between the two datasets, and the absence of technical replicates in the NEUROG2 experiment, the majority of NEUROG2 binding regions overlapped NEUROD2 sites, indicating that both TFs could operate on a shared subset of genomic regions (Figure 1C).

Secondly, in order to provide chromatin context to these binding events, we reprocessed single-cell ATAC-seq data obtained from the same developmental time and tissue (50)⁠, obtaining four groups of peaks representing different chromatin accessibility trajectories in neurodevelopment: (i) invariant (non-variable across cell types and pseudotime), (ii) falling (displaying maximum accessibility in NSCs and being subsequently closed), (iii) transient (made accessible in the course of neurodifferentiation and then closed in mature neurons) and, (iv) neuronal (opened in the course of neurodifferentiation and still accessible in mature neurons) (Figure 1B and Supplementary Figure S2A and B)

The intersection of the NEUROG2-enriched binding sites revealed a significant enrichment in falling trajectories (Fisher’s exact test, *P*= 1.89e-91; OR = 7.9), and to a lesser extent, transient trajectories (Figure 1D and Supplementary Figure S2C). Conversely, NEUROD2-enriched binding sites were highly enriched in neuronal and transient ATAC-seq regions (Fisher’s exact test, *P* ≤ 2.23e-308; OR = 9.56, *P* ≤ 2.23e-308; OR = 17.16, respectively) (Figure 1D and Supplementary Figure S2D). The subset of NEUROG2/NEUROD2 shared binding regions was even more enriched in transiently accessible chromatin regions (Fisher’s exact test, *P*= 2.06e-62; OR = 2.88), peaking in the period when these TFs exhibited some expression overlap (Figure 1D and Supplementary Figure S2E). We could observe that these highly dynamic regions enriched in NEUROD2-enriched and NEUROD2/NEUROG2 shared binding sites gain accessibility specifically in the lineage of excitatory projection neurons and not in other cell types present in the embryonic neocortex, including inhibitory neurons and microglial cells (Supplementary Figure S2F). Moreover, they are predominantly found in enhancers compared with promoters (Supplementary Figure S2G-I). Taken together, our results suggest that although NEUROG2 and NEUROD2 exhibit private binding sites indicative of specialized functions in NPCs and neurons, respectively, they may regulate a common subset of targets involved in transient functions in IPCs and early born neurons.

Another inference from our analysis is that both NEUROG2 and NEUROD2 binding tend to be associated with the chromatin trajectories that most closely mirror their expression patterns. With this in mind, we re-classified the ATAC-seq peaks based on the correlation of their accessibility along the neurodifferentiation axis with the expression of *Neurog2* and *Neurod2*, derived from matched transcriptomics data on the same cell populations (50) (Figure 1E). After ordering ATAC-seq regions based on this criterion, we divided them in 50 quantiles and studied the occupancy of NEUROG2 and NEUROD2 in those groups. This analysis confirmed our previous observation: top correlated peaks exhibited the highest proportion of binding sites of the corresponding factor (Figure 1E). Strikingly, 86% of the ATAC-seq peaks in the top 50th quantile most correlated with *Neurod2* expression were bound by NEUROD2, versus only 5.42% of the first quantile (15.87-fold enrichment). In the case of NEUROG2, although the absolute occupancy levels were lower than for NEUROD2, the difference between the top and bottom correlation quantiles was even more evident: 26.65% versus 0.11% (fold change of 242.72). We also sorted the peaks based on the correlation with the joint *Neurog2*+ *Neurod2* expression and, interestingly, the binding sites shared by NEUROG2 and NEUROD2 displayed the highest enrichment in the top correlated fraction of ATAC-seq peaks (fold change of 316.06 between the first and last quantiles) (Supplementary Figure S2K). Together, these results establish an association between the expression and DNA binding of NEUROG2 and NEUROD2 with a vast set of genomic regions exhibiting dynamic chromatin accessibility during neurogenesis and excitatory neuron differentiation.

### Chromatin accessibility and gene functions associated with NEUROD2 in the prenatal and postnatal neocortex

**Figure 2.**
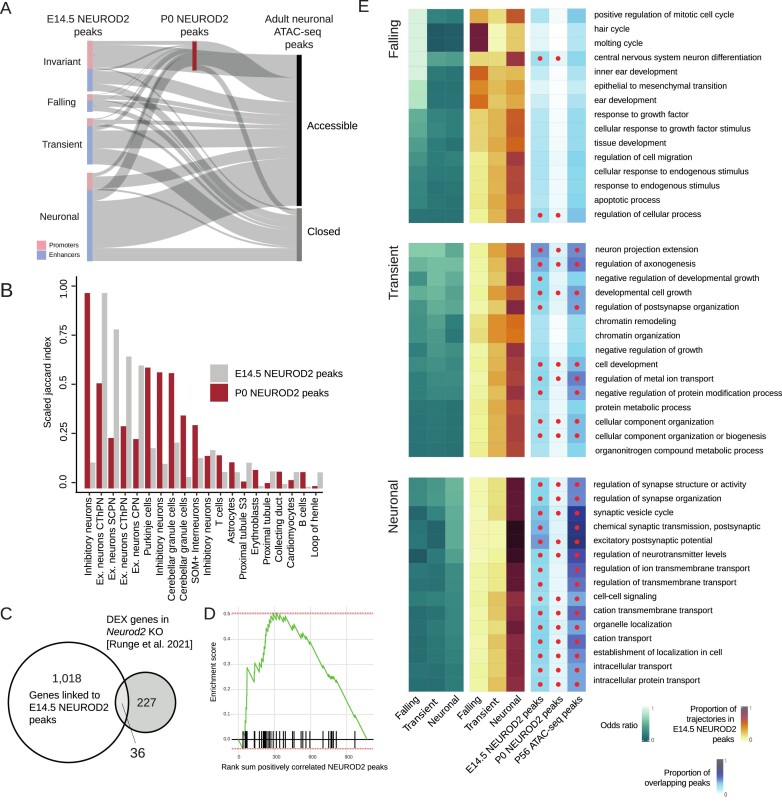
Biological functions associated with *Neurod2* in the prenatal and postnatal neocortex. (**A**) Sankey plot representing the relative number of peaks of the different trajectories which are bound or not by NEUROD2 in the P0 embryonic cortex, and that map to accessible chromatin regions in adult mouse cortical excitatory neurons. (**B**) Barplot displaying scaled Jaccard indices between NEUROD2 E14.5 and P0 peaks with regions accessible in various cell types of the mouse adult whole body. (**C**) Venn diagram depicting the intersection between genes linked with E14.5 NEUROD2 peaks and genes differentially expressed (DEX) upon *Neurod2* knockout in the adult mouse neocortex (21). (**D**) Gene set enrichment analysis of the DEX genes in the *Neurod2* knockout in the adult mouse neocortex ranked by their linked peak with maximum correlation with the expression of *Neurod2*. (**E**) Heatmaps showing (i) odd ratios of the top 15 gene ontology terms enriched in each of the ATAC-seq trajectories (left), (ii) proportion of trajectories among the NEUROD2 peaks positively linked to genes in each ontology term (middle), and (iii) fraction of peaks linked to genes in each ontology term intersecting with E14.5 and P0 NEUROD2 peaks, as well as with P56 excitatory neuron ATAC-seq peaks. Dots indicate statistical significance, as measured by a Fisher’s exact test (right).

The expression of *Neurod2* subsides postnatally but is not abolished, indicating its potential role in mature neurons (21). We set up to investigate the genomic regions targeted by NEUROD2 at E14.5 that remained targeted and/or accessible after birth. To achieve this, we utilized a NEUROD2 ChIP-seq dataset performed on the mouse cortex at postnatal day 0 (P0) (79) and a single-cell ATAC-seq dataset of the postnatal day 56 (P56) mouse cortex (59). While the majority of NEUROD2 peaks observed at E14.5 were undetected postnatally (Figure 2A), a significant proportion of these peaks remained accessible in postnatal excitatory neurons. This finding suggests a persistent reorganization of the chromatin during neurogenesis in NEUROD2-bound regions, or a sustained occupancy of Neurod factors in these genomic regions. The subset of NEUROD2 peaks which were also detected at P0, were primarily located in accessible regions associated with transient and neuronal categories (Figure 2A), highlighting a reduced set of positions that display continued NEUROD2 activity after birth.

To explore the cell-type specificity of NEUROD2-bound accessible regions at E14.5 and P0, we leveraged a single-cell atlas of chromatin accessibility encompassing 13 adult mouse tissues (60). Both sets of NEUROD2 peaks exhibited the highest enrichment in chromatin signatures associated with neurons (Figure 2B). However, E14.5 peaks displayed maximum enrichment in elements specific to excitatory neurons, with lower overlap with peaks associated with other neuron types. In contrast, P0 peaks exhibited substantial overlap with multiple classes of neurons, including inhibitory neurons, cerebellar granule cells and glutamatergic cortical projection neurons (Figure 2B). This disparity suggests that late NEUROD2 activity regulates functions shared by diverse neuronal classes.

To investigate the biological functions associated with NEUROD2 peaks in prenatal and postnatal neocortex, we employed gene-enhancer links computed by Noack *et al.* (50), which were⁠ based on the correlation between chromatin accessibility and gene expression within the same tissue and time point (80). Further supporting these predictions, we discovered a significant overlap between this set of targets and a group of 263 genes previously identified as DEX between the cortices of *Neurod2* KO and WT mice at P30 (Fisher’s exact test, *P*= 1.08 × 10–14; OR = 5.5) (21) (Figure 2D). Moreover, the set of 263 DEX genes were found to be enriched among the top ranking *Neurod2* targets, based on their correlation values between chromatin accessibility and gene expression (Ranksum test, *P*= 0.046) (Figure 2D). Through these analyses, we compiled a list of 36 putative *Neurod2* targets (Table 1), some of which are known to play critical roles in cell migration and axon development (WebGestalt over-representation analysis (ORA) false discovery rate (FDR) = 0.0056). Notably, genes such as *Rnd2* and *Tiam2*, each associated with six NEUROD2 peaks exhibiting a maximum correlation > 0.9 (Table 1), as well as *Nefm*, *Nrep* and members of the semaphorin-plexin signaling pathway were among the identified targets. Of particular interest is our observation regarding the gene *Rnd2*, with a critical role in neuronal migration, which was previously reported as a direct target of *Neurog2* (13). Intriguingly, our findings at E14.5 suggest that *Neurod2* exhibits a stronger association with *Rnd2* compared to *Neurog2* (Supplementary Figure S3A), which carries implications for our understanding of the roles reported for NEUROG2 in neuronal migration during late neurogenesis (13,15). In summary, our findings highlight a subset of strongly predicted *Neurod2* targets showing sustained expression alterations in postnatal KO mice, suggesting continued regulation by *Neurod2* and/or enduring effects of the *Neurod2* KO inherited from prenatal developmental phases.

**Table 1.** Top candidate targets of NEUROD2

Gene	# Neurod2 peaks	Maximum correlation	log_2_ fold change (FC) (KOvsWT)
Tiam2	6	0.915	−0.494
Nuak1	7	0.911	−0.473
Plxna4	4	0.911	−0.445
Ank3	3	0.883	−0.313
Tenm4	6	0.880	−0.473
Id2	3	0.867	−0.916
Abcd2	3	0.857	−0.496
Camk4	3	0.841	−0.314
Nrep	3	0.839	−0.355
Pdzrn3	4	0.831	−0.377
Nefm	3	0.823	−0.410
Smarca2	1	0.820	−0.298
Sema7a	6	0.814	−0.357
Lrrc55	2	0.799	−0.629
1110008P14Rik	2	0.784	−0.462
Dyrk2	4	0.777	−0.475
Sgtb	3	0.758	−0.284
Plch2	1	0.756	−0.307
Pcdh9	2	0.754	−0.311
Epha4	1	0.733	−0.436
Dgki	1	0.731	−0.397
Tmem178b	1	0.729	−0.346
Dock4	2	0.715	−0.351
Sort1	4	0.714	−0.453
Grin2b	2	0.707	−0.541
Adcy1	1	0.700	−0.461
Rnd2	6	0.930	0.403
Ly6h	1	0.881	0.303
Trp53inp1	2	0.880	0.454
Mc4r	2	0.855	0.618
Sla	4	0.847	0.645
Pde9a	6	0.836	0.451
Ociad2	3	0.804	0.395
Igsf21	3	0.775	0.376
Ddit4	1	0.739	0.380
Cdh13	1	0.724	0.509

Maximum correlation between target gene expression and ATAC-signal is shown. Differential expression in Neurod2 KO mice from Runge *et al.* (21) is indicated.

To unveil the distinct functional characteristics associated with *Neurod2* targets linked to ATAC peaks of different trajectories, we conducted a comprehensive gene ontology enrichment analysis on each category. The early peaks were primarily associated with broad early developmental functions, displaying minimal overlap with *Neurod2* targets and limited association with adult excitatory neuronal ATAC-seq peaks (Figure 2E). In contrast, ATAC peaks linked to transiently accessible regions exhibited significant enrichment in functions related to axonogenesis. Peaks associated with these genes displayed a high overlap with *Neurod2* putative E14.5 and P0 gene targets, as well as postnatally accessible regions. Given that previous mouse knock-out experiments have demonstrated the essential role of NeuroD genes in axonal fasciculation and the development of the corpus callosum (19), we conducted a detailed analysis of NEUROD2 targets among genes related to axonogenesis. We observed that NEUROD2 binds to regulatory elements associated with all classic axon guidance pathways, including Slit/Robo, Netrin/DCC, Netrin/Unc5, Semaphorin/Plexin and Ephrin/Eph receptors and multiple axon fasciculation molecules, such as Contactin 2 (*Cntn2*) and neurofascin (*Nfasc*) (Supplementary Figure S3B). Genes linked to ATAC-peaks with neuronal trajectories produced enrichments in terms related to synaptic transmission, showing significant overlap with both E14.5 and P0 NEUROD2 peaks, as well as peaks accessible in adult excitatory neurons. Collectively, these findings indicate that NEUROD2 mediates both temporally specific and sustained regulatory activities commensurate with chromatin remodeling throughout prenatal and postnatal cortex development.

#### Both NEUROG2 and NEUROD2 exhibit diverse E-box binding capabilities

Earlier studies have shown that variation in the central dinucleotide of the CANNTG E-box motif can affect the activity of bHLH factors in different ways: it can indicate differential usage of dimerization partners, confer a bHLH factor specificity with respect to the other members of its family, and affect both binding affinity to the DNA and transactivation strength of target genes (42,77)⁠. Consequently, we chose to explore the distribution of the types of E-boxes bound by NEUROG2 and NEUROD2 across different putative regulatory elements, stratified based on their ATAC signal versus expression correlation and trajectories of chromatin accessibility.

We first quantified the presence and centrality of E-boxes with all possible combinations of the internal dinucleotides by means of exact pattern matching. Since E-box core positions are palindromic and we disregard flanking positions, certain dinucleotide combinations are equivalent, resulting in a total count of 10 distinct types of E-boxes. It is essential to reiterate and clarify the nomenclature we employ for these E-boxes. We denote them based on the sequence in the forward strand of the two half-sites, as we believe this provides more immediate information about the individual binding of each monomer of the bHLH dimer, as we had previously proposed (77). For instance, an E-box with the CAGATG, or the equivalent CATCTG, sequence is referred to as CAT-CAG, indicating that one monomer binds a CAT half-site, while the other monomer binds a CAG half-site.

**Figure 3.**
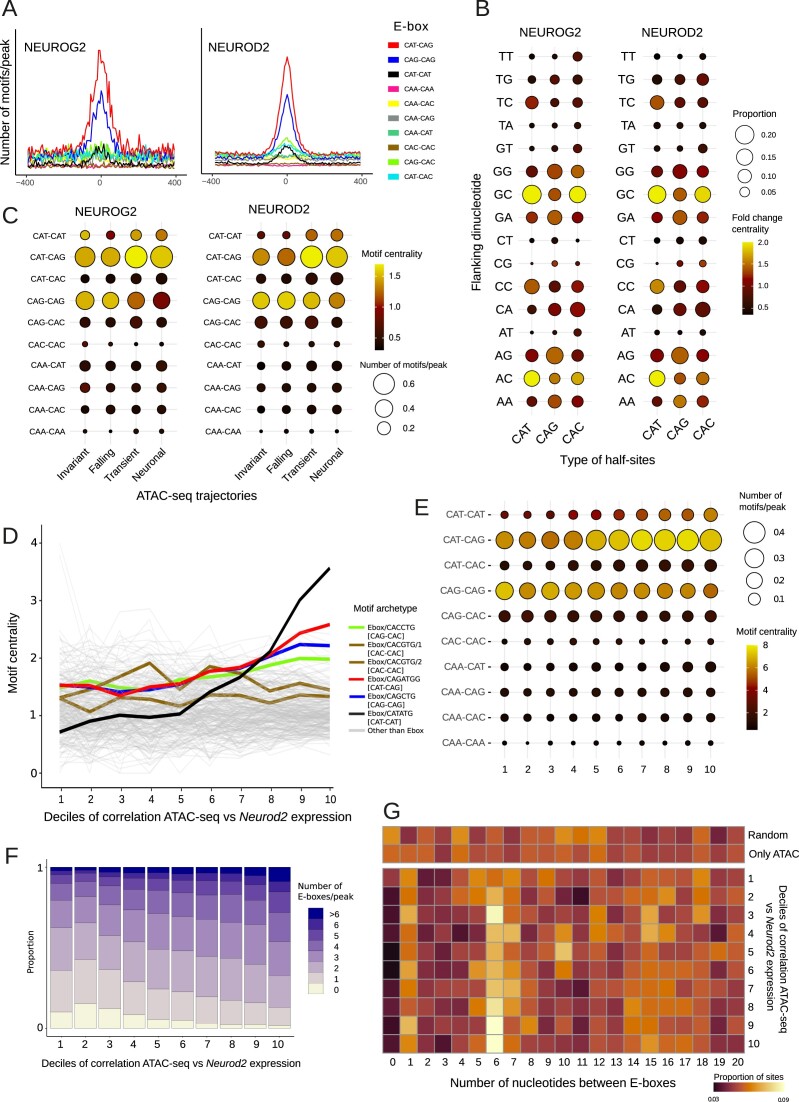
Motif grammar of proneural factors in the dynamic chromatin landscape of neurodifferentiation. (**A**) Number of E-box motifs per base pair and per peak around the summits of NEUROG2 and NEUROD2 peaks in the E14.5 mouse brain. (**B**) Dot plot indicating the proportion of dinucleotides in the flanking positions of half-sites of E-boxes identified 100 bp around the summit of NEUROG2 and NEUROD2 peaks, stratified for each half site (size). Color of the dots indicates the fold change of this proportion versus the corresponding proportion in motifs > 200 bp further away from the summit. (**C**) Dot plot representing the number of E-box per peak (100 bp around the summit) in the different ATAC-seq trajectories, as well as the fold change of this number versus motifs > 200 bp further from the summit. (**D**) Line plot indicating motif centrality (motifs 50 bp around the summit versus > 250 bp further) of all the archetypal motifs determined in Vierstra *et al.* (62) around the summits of the ATAC-seq peaks that intersect with NEUROD2 peaks, grouped by the correlation of their ATAC accessibility with the expression levels of *Neurod2*. (**E**) Centrality (motifs 50 bp around the summit versus > 150 bp further) of each E-box variant around the summits of the NEUROD2 peaks, binned by the correlation of the accessibility of the overlapping ATAC-seq peak with *Neurod2* expression levels. (**F**) Barplot displaying the proportions of NEUROD2 peaks encompassing different amounts of E-box motifs per peak, grouped by the correlation with *Neurod2* expression levels. (**G**) Heatmap indicating the proportion of nucleotides distances between pairs of E-boxes in NEUROD2 peaks, stratified by bins of increasing correlation between ATAC-seq peak accessibility and *Neurod2* expression levels. Spacing in random GC-content matched regions in the genome and ATAC-seq peaks with no overlap with NEUROG2/NEUROD2 peaks is also shown (top).

By counting and analyzing the distribution of each type of E-box around the summit of NEUROG2 and NEUROD2 peaks, we observed that CAT–CAT, CAT–CAG, CAT–CAC, CAG–CAG and CAG–CAC were enriched around peak summits (Figure 3A). Conversely, the remaining five types of E-boxes were randomly occurring in the DNA, with no centrality around the summits of the peaks. These findings align with previous evidence showing that CAA half-sites are non-preferred by all bHLH factors (81), and that CAC–CAC motifs are highly specific of the Myc subfamily of bHLH factors (66,77,82) and bHLH repressors, such as Hes2, which has been found enriched in inactive enhancers during cortical neurogenesis (50). We identified the CAT–CAG E-box as the most abundant (35.19% of all NEUROG2-bound E-boxes and 28.85% of all NEUROD2-bound E-boxes), followed by the CAG–CAG motif (21.99% and 19.76%). Although CAT–CAT motifs exhibited a lower absolute count and constituted only, 6.12% and 6.55% of all E-boxes within NEUROG2 and NEUROD2 peaks, respectively, they showed high centrality in their corresponding ChIP-seq peaks (Figure 3A). CAT–CAT E-boxes displayed comparatively lower enrichment at P0 (Supplementary Figure S4A), suggesting a temporally restricted role of this type of E-boxes, commensurate with *Neurod2* expression (Figure 1A).

#### E-box flanking nucleotides depend on the sequence of each half-site

We set up to identify possible biases among E-box flanking nucleotides. When we analyzed upstream flanking regions independently in each type of 5′–3′ oriented half-sites, we could note that each half-site was associated with different flanking dinucleotides (Figure 3B). For example, NEUROG2/NEUROD2-bound CAT half-sites showed enrichment in GC or AC preceding dinucleotides, whereas CAG half-sites were more associated with the AG dinucleotide. Previous structural studies indicated that the bHLH factors also make specific contacts with the flanking positions of the half-sites (81,83). Thus, the half-site-specific flanking bias observed here suggests the participation of different bHLH factors in the different half-sites, with each half-site being bound by a specific bHLH factor, which has also a preference towards binding some flanking dinucleotides over others. Finally, while previous *in vitro* studies observed variation in DNA binding affinity of E-proteins upon different methylation status of the CpG flanking the E-box (84), we observed no CG enrichment in the flanking positions of E-boxes bound by NEUROD2 and NEUROG2 (which are known to dimerize with E-proteins), indicating that the adjacent CpG methylation status is not a significant determinant of DNA binding of these factors *in vivo*.

#### E-boxes with CAT half-sites are preferentially enriched in chromatin regions correlated with Neurod2 expression

Each of the E-boxes examined displayed distinct enrichment patterns across the temporal trajectories of chromatin accessibility. Motifs containing a CAT half-site were more enriched in transient and neuronal trajectories, particularly within NEUROD2 peaks, in contrast to falling and invariant sites (Figure 3C). Similar results were obtained by calculating motif enrichments around the summit of NEUROD2-bound ATAC-seq peaks using a set of 286 non-redundant TF motif archetypes condensed by Viestra *et al.* (62) (Supplementary Figure S4B).

Since we have previously observed that NEUROG2 and NEUROD2 occupancy in ATAC peaks highly correlated with *Neurog2* and *Neurod2* expression (Figure 1E), we set up to measure how E-box counts, spacing and type preference varied with the strength of the association between ATAC signal and *Neurog2* or *Neurod2* expression. We sorted all ATAC-seq peaks bound by each TF into 10 quantiles based on their ATAC signal-expression correlation. Subsequently, we calculated the centrality around the summit of the ATAC peaks using the set of 286 archetypal TFs motifs from Vierstra *et al.* (62). Given that NEUROD2 binding sites are highly enriched in ATAC-seq regions that correlate with its expression, we anticipated a notable enrichment of E-box motifs in those highly dynamic regions commensurate to their relative abundance (Figure 3A), but our findings exceeded expectations. All motif archetypes, previously identified as potential targets for NEUROD2 binding, were enriched in the highly correlated regions. Surprisingly, CAT–CAT, a relatively minor motif in terms of overall frequency and attention in the literature, stood as the most centrally enriched motif, followed by CAT–CAG (Figure 3D). This observation is of utmost significance, as the centrality of motifs in chromatin regions serve as evidence for the pioneer capabilities of cognate TFs. When all 10 E-boxes were examined through exact pattern matching around the summit of the ChIP-seq peaks of NEUROD2, the preeminence of CAT–CAT E-boxes remained as the foremost associated with ATAC correlation to *Neurod2* expression, with CAT–CAG motifs showing a milder association (Figure 3E). Conversely, CAG–CAG motifs were more enriched in regions anti-correlated with *Neurod2* expression, and the rest of the motifs did not show apparent variation along the correlation axis. This enrichment of CAT–CAT E-box centrality within *Neurod2*-correlated sites was evident in both enhancers and promoter regions, even more pronounced in the latter (Supplementary Figure S4C). Employing the same strategy with NEUROG2-enriched peaks yielded no association between any E-box type and *Neurog2*-ATAC correlation (Supplementary Figure S4D), although CAT–CAT E-boxes also showed the highest centrality when ATAC-seq signal was correlated with the aggregated expression of Neurog2 and Neurod2 (Supplementary Figure S4E). Lastly, the centrality of CAT–CAT E-boxes was not observed in the ATAC-seq signal in other cell types, such as interneurons, microglial and mural cells (Supplementary Figure S4F), indicating that CAT–CAT E-boxes bound by NEUROD2 in the neocortex are associated with a chromatin remodeling effect specific to excitatory projection neurons. CAT–CAT E-boxes also exhibited central enrichment among the subset of ATAC-seq peaks that were correlated with *Neurod2* expression but not bound by NEUROD2 or NEUROG2 (Supplementary Figure S4G and H). Although other NeuroD factors, including the highly co-expressed *Neurod6*, could potentially explain this enrichment, the possibility of ChIP-seq false negatives in binding detection cannot be ruled out. To directly explore this possibility, we quantified the ChIP-seq signal in ATAC regions not bound by NEUROD2. We observed that ATAC regions exhibiting a high correlation with *Neurod2* displayed more NEUROD2 ChIP-seq signal (Supplementary Figure S4I). This finding suggests that subthreshold NEUROD2 peaks contribute to the centrality of CAT–CAT E-boxes within ATAC-seq peaks apparently not bound by NEUROD2.

Clustering of E-boxes in regulatory elements has been suggested to enhance both binding affinity to DNA and transcriptional activation of the genetic targets, as they facilitate cooperative binding of the bHLH factors to the DNA (42,85–90). We quantified the distribution of E-boxes in NEUROD2 peaks across the same *Neurod2*-ATAC correlation quantiles and found that the peaks that were more correlated with the expression of *Neurod2* contained a higher abundance of E-boxes (Figure 3F). We tested whether these clustered E-boxes were randomly distributed or if their spacing followed a specific pattern, as has been observed for TWIST1, which preferentially binds to 5 bp-spaced paired E-boxes as a homotetramer (91), or Ascl1 and Ascl2, which bind to closed chromatin through multiple E-boxes clustered by 10–15 bp (92). We found an over-representation of six nucleotide spacings between paired E-boxes within NEUROD2, but not NEUROG2, peaks, especially in regions highly correlated with *Neurod2* expression (Figure 3G). This 6-bp overrepresentation is lacking in peaks displaying the lowest correlation with *Neurod2* expression, random genomic regions with nucleotide composition or ATAC peaks with no NEUROG2/NEUROD2 ChIP-seq peaks (Figure 3G), suggesting intriguing functional implications.

Collectively, these findings suggest a specific grammar of NEUROD2 binding sites implicating clusters of motifs favoring certain spacing and types of E-boxes. Notably, although CAT–CAT motifs are less frequent than the other overrepresented E-boxes, namely CAT–CAG and CAG–CAG, they are by far the most enriched in highly dynamic regions in neurodifferentiation.

#### CAT–CAT E-boxes are associated with larger methylation changes during neurogenesis

Previous studies have underscored the significant role played by NEUROD2 and NEUROG2 in the demethylation of CpGs in intragenic and intergenic regions associated with neuronal genes during the process of neurogenesis. Noack *et al.* (48,50) integrated whole-genome CpG methylation profiling of sorted NSCs, IPCs and PNs with NEUROG2 and NEUROD2 ChIP-seq data of the E14.5 mouse cortex, concluding that both factors contributed to DNA demethylation throughout neuronal development. Conversely, Hahn *et al.*(47) attributed the demethylation specifically to NEUROD2, which they found overlapping half of the identified demethylated regions between PNs and NSCs. Interestingly, they associated this demethylation effect only with CAT–CAT and CAT–CAG motifs, and not with other E-box types. To shed light on this, we integrated the single-cell ATAC-seq and proneural factors ChIP-seq data described above with the CpG methylation dataset from Noack *et al.* (50).

**Figure 4.**
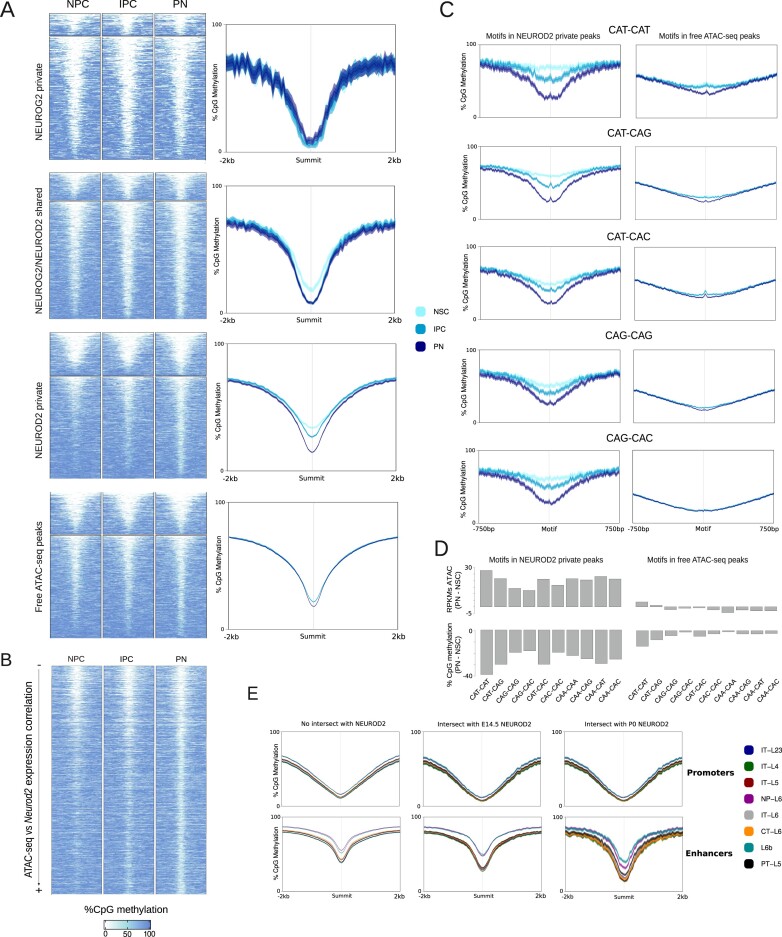
Demethylating effect of proneural factors’ binding during cortical neurodevelopment. (**A**) Heatmaps showing CpG methylation percentages in the main cell populations of the projection neuron lineage. Signal is measured in 4kb around the summits of the ATAC-seq peaks overlapping NEUROD2 or NEUROG2 private peaks, shared NEUROD2 and NEUROG2 peaks, or peaks not bound by NEUROD2 or NEUROG2 (left). On the right, the average percentage of CpG methylation is represented as lineplots, with ribbons indicating the standard error of the mean. (**B**) Heatmaps showing the percentages of CpG methylation around summits of ATAC-seq peaks intersecting NEUROD2 peaks sorted by the correlation of their accessibility with the expression levels of *Neurod2*. (**C**) Lineplots showing the average percentage of CpG methylation 1500 bp around different types of E-boxes within NEUROD2 peaks (left), and within ATAC-seq peaks that do not intersect with NEUROD2 (right). (**D**) Barplots representing the difference in percentage of CpG methylation measured 5 bp around each type of E-box between PN and NSC (bottom). The barplots on the top represent the same NSC-PN differences but using ATAC-seq RPKMs. (**E**) Average percentage of CpG methylation of different cortical projection neuron cell types in the adult mouse cortex in different subsets of regions.

This approach revealed that only those regions bound by NEUROD2 were significantly demethylated during neurodevelopment, especially from IPCs to PNs (Figure 4A). NEUROG2 was found to be associated with CpG demethylation only when its binding sites were shared with NEUROD2, albeit at a lower magnitude, while the demethylation observed in regions only bound by NEUROG2, or not bound by NEUROG2 or NEUROD2, was minimal (Figure 4A). However, regions bound by NEUROD2 exhibited varying degrees of demethylation commensurate to the initial CpG methylation levels in NSCs and chromatin accessibility dynamics (Supplementary Figure S5A, left). Transient and neuronal regions bound by NEUROD2 undergo the most pronounced demethylation, while invariant and falling regions appeared already demethylated in progenitor cells. With respect to peaks not bound by NEUROD2, those found within transient and neuronal enhancers also exhibited CpG demethylation, but to a lesser degree when compared with the NEUROD2-bound peaks of the same trajectories, indicating that the demethylation effects are more pronounced in the presence of NEUROD2 (Supplementary Figure S5A, right). The observed correlation between demethylation and chromatin accessibility dynamics was even more evident when we analyzed CpG methylation levels in regions sorted by the correlation between ATAC signal and *Neurod2* expression (Figure 4B).

Next, we measured CpG methylation changes in regions surrounding each type of E-box. We found that CAT–CAT, followed by CAT–CAG E-boxes, exhibited the strongest association with demethylation, whereas CAG–CAG and CAG–CAC E-boxes did not demonstrate association with demethylation, as they were predominantly located in demethylated regions in NSCs (Figure 4C and D). All these differences were more remarkable when comparing enhancer regions versus promoter regions, which tend to be epigenetically more static (Supplementary Figure S5B). Again, the CpG demethylation associated with each E-box mirrored chromatin accessibility dynamics: the motifs that were associated with a greater demethylation also were present in regions that gained more accessibility between NSC and PNs (Figure 4D and Supplementary Figure S5B). While E-boxes within regions not bound by NEUROD2 also reached similar methylation levels at PNs, these regions were already largely demethylated in NSCs, resulting in a minor transition which was also observed at the level of chromatin accessibility (Figure 4C and D).

Finally, to investigate the lasting stability of CpG methylation changes beyond birth, we assessed the percentage of CpG methylation in distinct subtypes of cortical excitatory neurons using single-cell methylation data from the adult mouse brain (93). Across all subsets of peaks, we observed substantial variations in methylation levels among neuron subtypes, with intra-telencephalic types consistently displaying the lowest levels (Figure 4E). Notably, both promoters and enhancers bound by NEUROD2 exhibited significantly lower CpG methylation levels in all neuron subtypes, in comparison to ATAC peaks not bound by NEUROD2 (Figure 4E). Interestingly, genomic regions containing NEUROD2 peaks detected at P0 displayed even lower methylation levels in adult neurons compared to those identified at embryonic E14.5 (Figure 4E).

In summary, through the stratification of genomic regions based on the intersection with NEUROG2/NEUROD2 peaks and specific E-box motifs, we observed substantial differences in the magnitude of CpG demethylation throughout neuronal differentiation, mimicking the aperture of the chromatin. We found NEUROD2-bound CAT–CAT, followed by CAT–CAG sites, to overlap regions exhibiting the largest demethylation changes, coinciding with the results by Hahn *et al.* (47). However, we also observed demethylation associated with other types of E-boxes, such as CAG–CAG and CAG–CAC. Regarding the potential role of NEUROG2 as driver of CpG demethylation proposed by Noack *et al.* (48,50), contrary to that presented by Han *et al.* (47), our analysis indicates that none of these observations can be concluded from the available data. The subset of NEUROG2-bound sites that are also bound by NEUROD2 appear to be the only sites that undergo demethylation, so the possible implications for NEUROG2 cannot be readily ascertained from the existing data. Moreover, those sites bound exclusively by NEUROG2 are already demethylated, so we cannot assess the demethylation potential for NEUROG2 on those regions.

#### HT-SELEX reveals a unique preference for CAT–CAT E-boxes for NEUROD2

Previous studies utilizing electrophoretic mobility shift assays showed that NEUROD2 and NEUROD1 bind to CAT–CAG E-boxes as heterodimers with the TCF4 factor, whereas they are not able to bind to this sequence as homodimers (32,40). Moreover, the proneural factor BHLHE22 exhibits homodimerization preference *in vitro* and a binding enrichment in CAT–CAT E-boxes *in vivo* (27). This homodimer preference for CAT–CAT E-boxes extends to other non-proneural members of the same bHLH subclass, such as TWIST1 (94) or OLIG2 (95).

**Figure 5.**
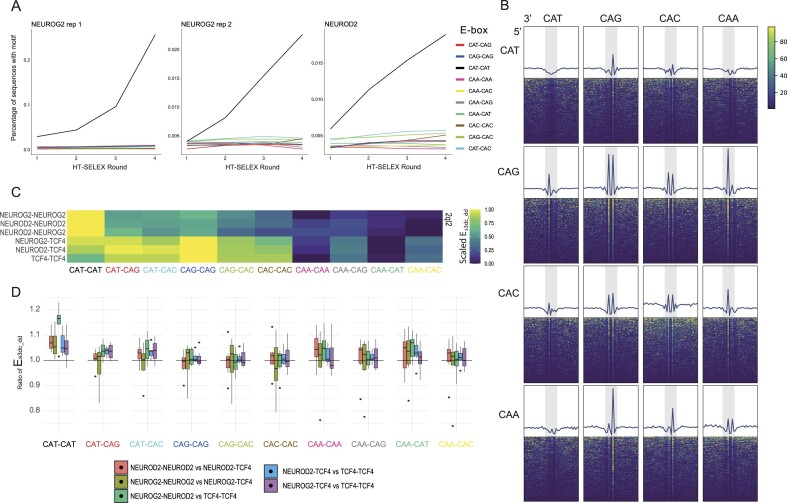
Analysis of the differential binding affinity of proneural factors among E-box variants. (**A**) Lineplot representing the fraction of sequences that contain each E-box variant in the successive rounds of HT-SELEX. (**B**) Footprinting analysis showing HINT-normalized transposase cut frequency in aggregated (lineplot, up), or in individual peaks (heatmap, down), around all possible strand-oriented CANNTG hexanucleotides in regions bound by NEUROD2 (**C**) ModCRE calculated binding energies between various combinations of NEUROD2, NEUROG2 and TCF4 dimers in each type of E-box using the PDB model 2ql2 as template. Heatmap displays row-scaled normalized binding energies. (**D**) Boxplot plot showing the distribution across different PDB structures templates of the normalized binding energies for various combinations of NEUROD2, NEUROG2 and TCF4 dimers in each type of E-box.

All this previous evidence together suggests that NEUROD2 binds to the CAT–CAT as a homodimer, and to CAT–CAG as a heterodimer with E-proteins. However, it remains a plausible scenario that NEUROD2 dimers may exhibit suboptimal binding affinity to non-CAT–CAT E-boxes. High-throughput *in vitro* assays, such as HT-SELEX, provide an ideal system to determine bona fide TF affinities among all possible sequences tested simultaneously, ultimately identifying the highest affinity sequences bound by the TF (96). Jolma *et al.* (66) performed the HT-SELEX assay with a large number of TFs, including bHLH factors in their homodimeric form. They derived sequence logos indicating the consensus motif most enriched in the HT-SELEX experiment, indicating a preference of NEUROD2, NEUROG2 and the rest of proneural factors for the CAT–CAT E-box (66,77,97). However, in these analyses the relative enrichment of each type of E-box was not explicitly interrogated. To that aim, we reanalyzed the successive HT-SELEX rounds obtained for NEUROG2 and NEUROD2 and measured the proportion of sequences containing each type of E-box in each round. In each round of HT-SELEX, an increase in the enrichment of the motif bound by the TF is expected with respect to the previous round. We observed, both for NEUROG2 and for NEUROD2, that the number of sequences containing the CAT–CAT motif increased in each round of the experiment, whereas the other variants of the E-box remained constant (Figure 5A). This indicates that NEUROD2 homodimers bind exclusively CAT–CAT E-boxes, at least in this *in vitro* system. These findings support the premise that CAT–CAT and CAT–CAG E-boxes, when occupied by NEUROD2 or NEUROG2, are operated in different partner configurations.

#### CAT half sites carry a distinct footprinting signature

We reasoned that if E-box usage could explain differential dimer composition, this might be reflected in differential DNA cleavage footprints among E-box types. To explore this, we conducted a footprinting analysis using the single-cell ATAC-seq signal around the different E-box types present in peaks bound by NEUROG2 and NEUROD2. Transposase cleavage frequencies were computed and corrected for expected cleavage bias with HINT-ATAC (98). When we aggregated the corrected cleavage frequency around the various E-box types within the NEUROD2 and NEUROG2 peaks, we discovered distinct footprinting signatures (Figure 5B and Supplementary Figure S6A). Notably, we only observed a decrease in transposase cleavage frequency in the CAT half-sites, and an increase in the other half-site sequences. These results might suggest that the CAT half-site is bound with more affinity by the TF, reducing the insertion of the transposase, whereas the other half-sites are bound with less affinity and are thus more accessible for the transposase. In line with this, a study analyzed the insertion of the DNase around the binding sites of the CAC–CAC E-boxes bound by the bHLH USF1 factor and found a footprinting pattern similar to the one we observed here for the CAC, CAG and CAA half-sites, with a peak of insertion frequency in the center of the half-site (99). When they compared this footprint with the crystal structure of USF1 bound to DNA, they found that the nucleotides of the motif showing less cleavage frequency were the ones that made less contact with the TF in the crystal structure, so they interpreted that these nucleotides were less protected and thus more prone to be contacted by the transposase. Applying this reasoning to our data, we can infer that the central ‘A’ nucleotide in the CAG, CAC and CAA half-sites show reduced interaction with the TFs, whereas in the case of the CAT half-site, all nucleotides are protected. Interestingly, the crystal structure for the NEUROD1-TCF3 dimer binding to the CAT–CAG motif, resolved by Longo *et al.*(83), aligned with this inference. In that structure, NEUROD1 contacts the entire phosphate backbone of its CAT half-site, while TCF3 does not contact the phosphate between the ‘A’ and the ‘G’ of its CAG half-site. These insights further support our hypothesis regarding the role of distinct dimer compositions in influencing E-box usage, as they show that in the heterodimer configuration CAT half-sites tend to be bound by the proneural factor whereas CAG half-sites are contacted by E-proteins (83).

#### Prediction of differential TF-binding affinity among E-boxes and bHLH combinations through homology-based modeling

To delve deeper into the DNA binding preference of different combinations of proneural factors and co-existing E-proteins during neurogenesis, we employed a recent structure homology modeling approach called ModCRE (100). Using this method, we systematically investigated the TF–DNA binding affinities for each type of E-box in various bHLH homodimers and heterodimers. ModCRE revealed higher protein–DNA binding affinities for CAT–CAT E-boxes in NEUROD2 and NEUROG2 homodimers (or NEUROD2–NEUROG2 heterodimers), and for CAT–CAG and CAG–CAG E-boxes in TCF4 homodimers or NEUROG2/NEUROD2-TCF4 heterodimers (Figure 5C). E-boxes containing CAA half-sites exhibited the lowest binding affinities regardless of the accompanying half-site. The observation of a higher affinity for CAT–CAT E-boxes of proneural homodimers compared with dimers containing E-proteins, or proneural/E-protein heterodimers compared with E-protein homodimers, remained largely consistent across all seven template structures used for the initial modeling of the different dimers (Figure 5D). We also quantified TF–DNA binding affinity at the nucleotide level, within each E-box. NEUROD2 and NEUROG2 homodimers, as well as NEUROG2–NEUROD2 heterodimers, exhibited a higher affinity for the internal dinucleotides of the CAT–CAT E-boxes when compared to TCF4-containing structures (Supplementary Figure S6B). However, when comparing different E-boxes, these proneural homodimers also showed similar affinities with the internal dinucleotide of CAG–CAG E-boxes, in contrast to the relatively lower affinity observed in the global structure compared to CAT–CAT E-boxes (Figure 5C and D). Nonetheless, the nucleotide affinities towards the CAG–CAG motif were lower in absolute terms when compared to those exhibited by the TCF4 homodimer. TCF4 homodimers displayed a higher affinity for internal dinucleotides of CAG–CAG E-boxes, and a lower affinity in CAT–CAT E-boxes (Supplementary Figure S6B). In CAT–CAG E-boxes, TCF4 homodimers showed a higher affinity for the G nucleotide, as compared to the T nucleotide (Supplementary Figure S6B). Finally, we explored interaction differences between residues within the basic DNA-binding domain of each TF and CAT–CAT or CAG–CAG E-boxes. While most residues showed similar interaction scores with both sequences, differences surfaced in the last amino acid of the 13 residues constituting the basic domain, a defining variable position in the bHLH class (77,101). Specifically, the Val580 of TCF4 displayed better interaction with CAG–CAG, whereas Met135 or Met125, present in the corresponding position within the sequence of NEUROD2 and NEUROG2, respectively, interacted with higher score with the CAT–CAT E-box sequence (Supplementary Figure S6C and D).

Taken together, the reanalysis of our *in vitro* binding assays, footprinting analysis and structural prediction collectively suggest a univocal relationship between E-box usage and dimeric composition of proneural factors and E-proteins.

#### E-box usage in chromatin reconfiguration after induction of proneural factors *in vitro*

**Figure 6.**
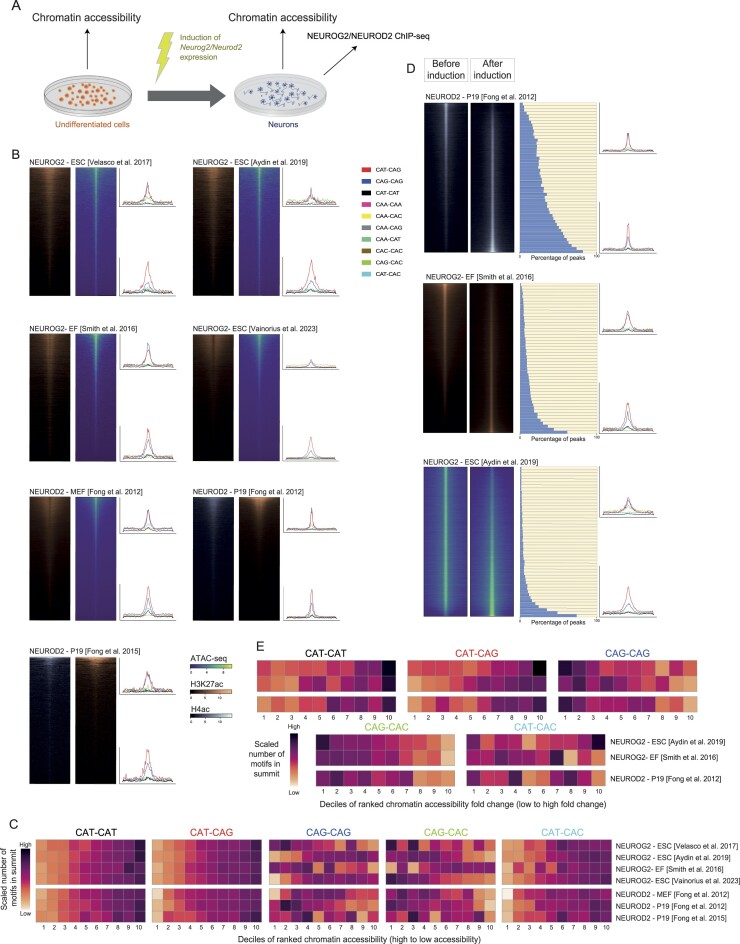
(**A**) Schematic representation of an *in vitro* experiment involving the induction of a proneural factor, chromatin accessibility profiling and the identification of proneural factors’ binding sites. (**B**) Heatmaps showing chromatin accessibility signal measured as RPKMs in regions spanning 6kb around summits of NEUROD2 or NEUROG2 peaks and sorted by chromatin accessibility signal, from highly accessible to less accessible for each induction study. Next to each study, two density plots are shown depicting the number of E-box motifs per base pair and per peak around the summits of NEUROG2 or NEUROD2 peaks of the first and bottom deciles of the peaks sorted by chromatin accessibility. (**C**) Heatmaps representing the number of each type of E-box in regions within each decile of chromatin accessibility scaled across all deciles in each E-box. Each row represents one of the induction studies included in B). (**D**) (Left) Heatmaps representing chromatin accessibility before and after proneural factor induction in three induction studies. (Middle) Proportion of sites bound by NEUROG2 or NEUROD2 in each of 50 quantiles of regions sorted by chromatin accessibility fold change pre- versus post-induction. (Right) Density plots are shown depicting the number of E-box motifs per base pair and per peak around the summits of NEUROG2 or NEUROD2 peaks of the first and bottom deciles of the peaks sorted by the fold change of chromatin accessibility pre- versus post-induction. (**E**) Heatmaps representing the number of each type of E-box in regions within each decile of pre- versus post-induction fold change of chromatin accessibility scaled across all deciles in each E-box. Each row represents one of the induction studies included in D).

Ectopic induction of TF expression, combined with chromatin accessibility and TF binding measurements, provides a controlled experimental framework to assess the impact of a TF on chromatin reconfigurations and differential motif usage (Figure 6A). To explore this idea, we reanalyzed six NEUROD2 and NEUROG2 induction experiments across diverse cell types, including embryonic stem cells (ESCs), P19 carcinoma cells and embryonic fibroblasts. By combining post-induction NEUROG2 and NEUROD2 ChIP-seq data with pre-induction chromatin accessibility assays, we categorized binding sites based on their chromatin accessibility and compared the enrichment of the different E-box variants in those regions. We observed that both factors can bind inaccessible chromatin, and that most inaccessible regions exhibited a higher enrichment of CAT–CAT motifs, followed by CAT–CAG motifs, compared to highly accessible regions (Figure 6B and C).

Three of these studies profiled chromatin accessibility both before and after induction, alongside NEUROD2 or NEUROG2 ChIP-seq data, enabling a direct quantification of the TF’s impact on chromatin changes. We analyzed the fold change in chromatin accessibility across all peaks pre- and post-induction, categorizing them into quantiles based on fold change and assessing their overlap with NEUROD2 or NEUROG2 peaks. Importantly, proneural factor binding strongly correlated with the degree of epigenetic reconfiguration following *NEUROG2* or *NEUROD2* induction, particularly in regions transitioning from closed to highly accessible chromatin (Figure 6D and E). Consistent with our findings in primary mouse cortex data (Figure 1), CAT–CAT E-box motifs, followed by CAT–CAG E-boxes, displayed the highest relative enrichment in genomic regions undergoing significant epigenetic changes (Figure 6D and E).

#### CAT–CAT E-boxes exhibit the highest level of sequence conservation

**Figure 7.**
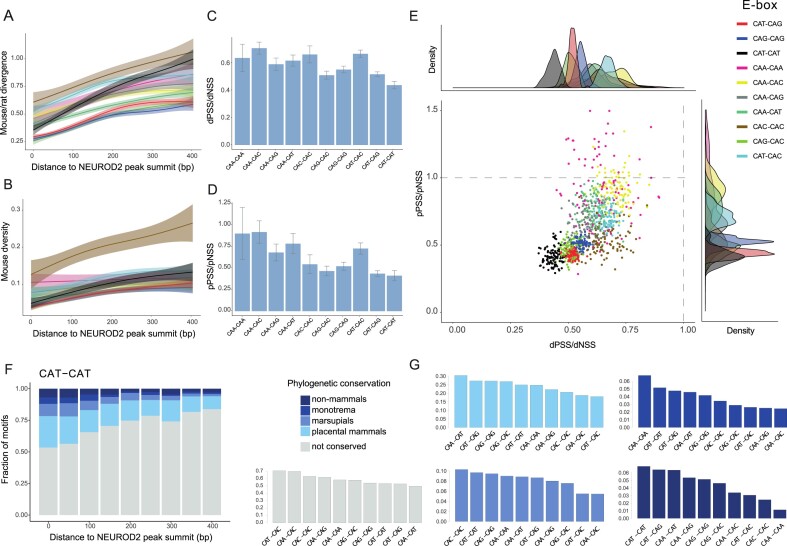
Sequence conservation of E-box motifs within NEUROD2 peaks. (**A**) Line plots displaying the number of mouse-rat substitutions within E-boxes in bins of increasing distance to NEUROD2 peak summits. Lines denote mean number of substitutions, and ribbons, standard deviation from the mean. (**B**) Line plots showing number of SNPs within E-boxes in bins of increasing distance to NEUROD2 peak summit. (**C**) Barplot showing the ratio of the pPSS and pNSS in each E-box type in NEUROD2 peaks. (**D**) Barplot showing the ratio of the dPSS and dNSS in each E-box type in NEUROD2 peaks. (**E**) Scatter plot showing pPSS/pNSSand dPSS/dNSS ratios in subsets of E-boxes of each type found in the summit of NEUROD2 peaks. (**F**) Proportion of phylogenetic depths of CAT–CAT E-boxes at bins of increasing distance to NEUROD2 peak summits. (**G**) Barplots showing proportion of E-box variants among E-boxes at various assessed phylogenetic depths.

We next investigated the level of sequence conservation within the entire set of E-boxes bound by NEUROD2 and NEUROG2. We conducted measurements of divergence by counting substitutions against the rat genome. In parallel, we assessed the sequence diversity within these E-boxes by harnessing a single nucleotide polymorphism (SNP) panel derived from 154 whole-genomes obtained from wild mice (74). We noticed a clear relationship between both divergence and diversity and the distance of E-boxes from the NEUROD2 peak summit (Figure 7A and B, respectively). We then compared divergence and diversity values between those E-boxes proximal to the peak summit (50 bp around the summit), that we called putatively selected sites (PSS), and E-boxes in regions distal to the summit (200–400 bp), which we called non-selected sites (NSS). Our analysis of the PSS/NSS ratios revealed that CAT–CAT E-boxes display the most pronounced change, followed by CAT–CAG E-boxes, a trend observed both in terms of divergence and diversity (Figure 7C and D). In general, observations were consistent between NEUROD2 and NEUROG2 in terms of divergence, albeit the higher variation levels in the later due to the smaller number of NEUROG2 peaks analyzed and variants overlapping those peaks (Supplementary Figure S7A and B). We contrasted diversity and divergence in PSS and NSS under the Macdonald–Kreitman framework (Figure 7E) using random subsets of E-boxes of the same type. Except for CAA–CAA and CAA–CAC E-boxes, all types reside in the quadrant denoting purifying selection (i.e. pPSS/pNSS < 1 and dPSS/dNSS < 1). Together, these analyses identified CAT–CAT E-boxes as the most highly constrained motif within contemporary mice populations that extends through to the period of their evolutionary divergence from rats.

To further explore the depth of sequence conservation among different E-box types, we used MULTIZ alignments of 60 species of vertebrates and determined the emergence of each E-box within the species tree (see the ‘Materials and methods’ section). Once again, we observed a clear relationship between the depth of phylogenetic conservation and the proximity to the NEUROD2 peak summit (Figure 7F and Supplementary Figure S7C). Focusing specifically on E-boxes situated near the summit (100 bp around the summit), we found that CAT–CAT E-boxes were the most conserved across vertebrates, extending beyond mammals, and the second most highly conserved until the time of the split with monotremata (i.e. platypus) (Figure 7G). Following closely were other CAT-containing E-boxes, such as CAT–CAG and CAT–CAA, which also exhibited considerable levels of conservation (Figure 7G).

In summary, our results indicate that, although CAT–CAT E-boxes constitute only a limited portion of the overall E-box binding repertoire for NEUROD2 and NEUROG2, they display the most pronounced evolutionary constraint, as evidenced by measures of diversity, divergence and phylogenetic depth.

#### Epigenetic reconfiguration is driven by homodimeric proneural factors

The concentration of proneural factors and E-proteins is likely to play a pivotal role in determining the specific dimer composition that forms during neuronal differentiation, and consequently, E-box usage. Notably, E-proteins are expressed at significant levels by the time *Neurod2* starts to be expressed (Figure 1A). Furthermore, heterodimers between proneural factors and E-proteins seem to be more stable and exhibit longer half-lifes (e.g. 5 h compared to 30 min of NEUROG2/E-protein heterodimers and NEUROG2 homodimers, respectively) (26,102). Therefore, it is reasonable to think that when proneural factors begin to be expressed in early phases of neurogenesis, they predominantly form heterodimers with the highly abundant E-proteins, and it is not until later in neurodifferentiation when enough proneural factors accumulate, so as a gradual shift in the stoichiometry of E-proteins and proneural factors begins to favor the formation of proneural homodimers. As we have previously seen, proneural factors show a preference towards CAT–CAG and CAG–CAG motifs when forming heterodimers with E-proteins, and to CAT–CAT when making homodimers. We also inferred that the CAT–CAT motif is the one bound with more affinity, followed by the CAT–CAG, and then the CAG–CAG motif.

**Figure 8.**
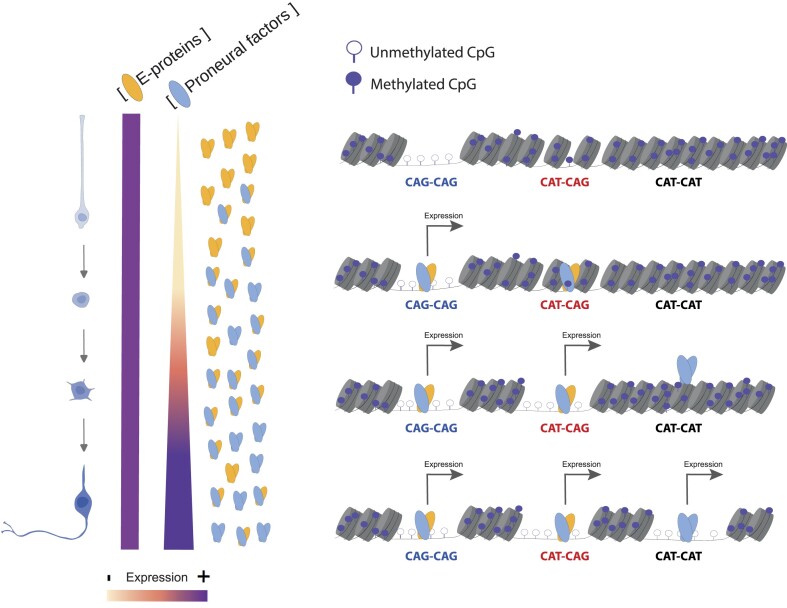
Schematics representing the properties of CAT–CAT E-boxes and the relationship between CAT–CAT occupancy and the homodimeric or heterodimeric action of proneural factors as a function of their relative concentration within the nucleus.

As shown in Figure 3C and E, CAG–CAG motifs are prominently enriched in regions already open in progenitor cells, whereas CAT–CAG motifs are prominently enriched in regions gaining accessibility during neurodifferentiation. CAT–CAT motifs are enriched in the most dynamic fraction among those regions, i.e. those undergoing the most extensive remodeling. Connecting all these previous observations, we can envision a model whereby in early stages of neurogenesis, proneural factors bind as heterodimers with E-proteins to regions that are already accessible and demethylated through the low-affinity CAG–CAG motifs (Figure 8). An increased concentration of heterodimers would allow them to outcompete histones for DNA binding and thus access the partially occluded CAT–CAG motifs, leading to the remodeling of chromatin around them and the demethylation of the surrounding CpGs. Subsequently, proneural factor homodimers would bind to the high-affinity CAT–CAT motifs present in highly packed regions that require extensive chromatin remodeling to be epigenetically active. As a final remark, our analyses support the hypothesis of the dynamic composition of proneural dimers. At early stages, NEUROG2–NEUROG2 homodimers would form, followed by NEUROG2–NEUROD2 heterodimers, and finally NEUROD2–NEUROD2 homodimers. Additionally, NEUROD1 and NEUROD6 could also potentially contribute to the formation of the CAT–CAT-binding proneural dimers.

An illustrative example of the explanatory power of our model is provided by the study of the promoters of *Neurod1*, induced at early stages of neurodifferentiation, and Delta (Dll1), which regulates the NPC state maintenance in surrounding cells. It has been demonstrated that heterodimerization with E-proteins enhances the ability of NEUROG2 to activate the expression of the *Delta* gene (26), while reducing both the ability of NEUROG1/2 to activate the *Neurod1* gene and their binding strength to its promoter (26,39,103). The study of E-box content and chromatin dynamics of the *Delta* and *Neurod1* promoters can shed light on these previous observations (Supplementary Figure S8A). We can observe that the Delta promoter, which is highly accessible in progenitor cells, contains four CAA–CAT and a single CAT–CAG motif overlapping NEUROG2/NEUROD2 peaks, while the proneural binding sites on the *Neurod1* promoter, which is remodeled to gain chromatin accessibility during neurodifferentiation, encompass a CAT–CAT, a CAT–CAG, a CAT–CAC and a CAC–CAC E-boxes. Thus, in early neurodifferentiation, NEUROG2/NEUROD2 would bind as heterodimers with E-proteins in the already accessible Delta promoter using the CAT–CAG motif. Subsequently, increasing concentration of proneural factors would facilitate the formation of proneural homodimers, initiating the interaction with the CAT–CAT motif in the *Neurod1* promoter and triggering its chromatin remodeling. This heterodimer to homodimer temporal switch subsequently activates different biological processes controlled by Delta and Neurod1, i.e. promoting maintenance of neuronal progenitors and neurodifferentiation, respectively, nicely illustrating that the timing of E-box activation can be leveraged in neurodevelopment to temporally regulate functions that need to be activated sequentially.

This model can help to reconcile the two apparently contradictory observations indicating that both the induction (39) and repression (35) of E-proteins results in impaired neuronal differentiation. We propose that while elevated levels of E-proteins dampen the formation of proneural homodimers, crucial for binding CAT–CAT motifs in region requiring chromatin remodeling, a low concentration of E-proteins compromise the formation of proneural/E-protein heterodimers, hindering the ability of proneural factors to act upon the abundant CAT–CAG and CAG–CAG E-boxes which are also required in neurodifferentiation. The model establishes that precisely regulated relative levels of E-proteins and proneural factors are necessary to cover their binding landscape and associated targets.

## Discussion

The TFs NEUROG2 and NEUROD2 have been postulated as critical orchestrators of the genetic cascades that drive cortical neurodevelopment. To shed light on the mechanisms through which they activate those transcriptional programs, we integrated ChIP-seq datasets of NEUROG2 and NEUROD2 generated in the mouse embryonic cortex with time- and tissue-matched multi-omics data. By doing so, we found that the occupancy of both NEUROG2 and NEUROD2 is most prominently enriched in genomic regions displaying a trajectory of chromatin accessibility highly correlated with the expression levels of these TFs during neurogenesis. NEUROG2 binding is highly biased towards dynamic regions whose accessibility peaks in IPCs and nascent neurons, whereas NEUROD2 preferably binds to regions whose accessibility peaks in nascent and migrating neurons, occupying up to 80% of those regions. Looking for further evidence supporting the involvement of NEUROG2 and NEUROD2 in chromatin remodeling during cortical development, we analyzed the centrality of motifs within those dynamic ATAC-seq peaks, revealing that the ‘CANNTG’ E-box motifs characteristic of these factors were the most centrally enriched in regions transiently open specifically in the glutamatergic neuronal differentiation. This E-box centrality was found most prominent in regions whose accessibility correlated with the expression of Neurod2, providing support for the pioneer capabilities of this TF. We found a striking enrichment in the CATATG motif in these regions, followed by CAGATG and CAGCTG, which we denote as CAT–CAT, CAT–CAG and CAG–CAG, respectively. Re-analysis of induction experiments comparing chromatin accessibility before and after induction further highlights CAT–CAT motifs as being prominently enriched in regions experiencing the largest fold changes in accessibility. We also found that the CpGs around these motifs were demethylated in the course of neurodevelopment, in a manner that was proportional to the extent of the aperture of the chromatin. Importantly, we found that the CAT–CAT E-boxes were particularly conserved in evolution, both in terms of nucleotide polymorphism and divergence, followed by CAT–CAG, CAG–CAC and CAG–CAG E-boxes.

Our re-examination of HT-SELEX data and footprinting analysis coupled with structural modeling, provides compelling evidence that homodimers of NEUROG2 and NEUROD2 exhibit a marked preference for binding to CAT–CAT E-boxes whereas CAT–CAG E-boxes are bound by heterodimers formed in conjunction with E-proteins. Thus, by connecting observations derived from the reanalysis of functional genomics and *in vitro* datasets, complemented with structural analyses, we propose that the variation in E-box usage reflects different proportions of homodimeric and heterodimeric forms of the NeuroD factors and E-proteins. We also hypothesize that the NeuroD homodimer becomes more dominant in highly dynamic regions that gain accessibility during neuronal development.

Our approach, which involves classifying binding sites based on the correlation between ATAC signal and TF expression, has proven to be an effective strategy for extracting functional insights from TF ChIP-seq data. Notably, NEUROD2 occupancy exhibited a strong positive correlation with this measure, as did other grammatical features of the motifs, such as clustering and spacing. This approach holds promise for broader application across other TF and cell types, enabling the identification of key regulators of chromatin reconfiguration across various lineages, and their underlying DNA-binding mechanisms.

The stratification of NEUROD2 peaks based on ATAC-seq trajectories helps clarify the interpretation of previous studies. For example, Fong *et al.* found that CAT–CAG motifs exhibit larger transactivation of NEUROD2 targets in P19 cells, and the gene targets regulated by these E-boxes were found to be more associated with neuronal functions, all in comparison to the CAG–CAG motifs (42)⁠. In addition, Hahn *et al.* suggested that only by binding to CAT–CAT and CAT–CAG motifs, but not CAG–CAG and other E-boxes, was NEUROD2 capable of mediating CpG demethylation during neurogenesis. Both studies ascribed this differential functionality of NEUROD2, contingent on E-box usage, to changes in the protein′s structure induced by its interaction with the DNA. Fong *et al.* interpreted that, by binding to CAT–CAG motifs, NEUROD2 acquired a certain 3D structure favoring the recruitment of coactivators of the expression; Hahn *et al.* suggested that CAT–CAT and CAT–CAG motifs would enable NEUROD2 to acquire the structure needed to recruit the demethylating TET2 enzyme (47). Fong *et al.* suggested that NEUROD2 is largely restricted to accessible chromatin (42). However, their testing of NEUROD2’s ability to access closed chromatin focused solely on its binding to the CAG–CAG motif. According to our observations, this is the motif NEUROD2 predominantly uses to target accessible chromatin. On the other hand, Fong *et al.* showed that NEUROD2 is able to induce histone 4 acetylation around its binding sites, which is a mark of chromatin accessibility, and they suggested that this acetylation is induced equivalently at CAT–CAG and CAG–CAG binding sites. However, our re-examination of their results, along with the re-analysis of additional datasets profiling chromatin accessibility changes induced by NEUROG2 or NEUROD2, reveals greater chromatin reconversion at CAT–CAT, and to a lesser extent CAT–CAG, binding sites compared to CAG–CAG sites. Considering our results, we hypothesize that the differential binding affinity for different E-boxes, rather than E-box-dependent conformational changes induced in NEUROD2, underlie the functional distinctions observed among these specific E-boxes.

Indeed, both our NEUROD2 and NEUROG2 footprinting analyses, together with the study of the crystal structure derived by Longo *et al.* for the NEUROD1-TCF3 dimer bound to the CAT–CAG motifs, indicate that the proneural-bound CAT half-sites are contacted more tightly than the E-protein-bound CAG half-site. Consequently, we deduce a higher binding affinity in CAT–CAT motifs than in CAT–CAG motifs, with an even more pronounced difference compared to CAG–CAG motifs. A higher TF–DNA affinity implies a prolonged dwell time on the DNA, which enhances the ability of TFs to compete and displace histones (104,105), and at the same time, it also boosts their capacity to recruit cofactors required for chromatin remodeling and transcriptional activation (71,106,107). It is thus reasonable to assume that the TET2 enzyme may also benefit from increased NEUROD2 dwell time. As an illustrative example of this hypothesis, Li *et al.* found that NEUROG2 homodimers exhibited stronger binding to the *Neurod1* promoter and higher transactivation capacity of *Neurod1* compared to NEUROG2/E-protein heterodimers (26). Given our observation that the *Neurod1* promoter encompasses a CAT–CAT and a CAT–CAG E-box, it is plausible that the observed differences in binding strength and transactivation correspond to NEUROG2 homodimer binding with more affinity the CAT–CAT E-box than the NEUROG2-E-protein heterodimer its cognate CAT–CAG E-box. As an additional remark, we observed clustering of E-boxes in highly dynamic regions gaining accessibility in neurodevelopment. This observation is in line with our affinity hypothesis, since it has been observed that clustering of E-boxes enhances both DNA binding and transcriptional activation (42,77,85–90).

Our study supports the conclusion that proneural factors function as pioneer TFs that access closed chromatin and remodel it. This notion contradicts the predictions of a previous general model proposed by Soufi *et al.* (108), which states that pioneer factors are able to co-bind DNA with nucleosomes efficiently through the use of partially unfolded or short alpha helices, requiring short or degenerate DNA motifs. Since NeuroD factors bind to DNA with long fully folded alpha helices that contact the entirety of their DNA motif, Soufi *et al.* predicted that these factors would be incapable of binding to closed chromatin. Regarding motif degeneracy, the other requirement of the model, Soufi *et al.* indicated that NeuroD’s preferred motif is a fixed CAG–CAG E-box with no degenerate positions (108). However, our observations concerning NEUROG2 and NEUROD2, adding to previous findings in this and other NeuroD factors (17,42,109), clearly establish the CAG–CAG motif as one of many possible E-boxes recognized *in vivo* and *in vitro* by NeuroD factors. Moreover, several studies where ectopic expression of NeuroD, and their closely related Neurogenins were induced, revealed competence to engage closed chromatin and remodel it, provoking the conversion of different cell types into neurons, a notion that has been confirmed and expanded in the present study by re-analyzing various induction datasets (42,68,69,109,110). All this evidence being considered, we deem the statement that NeuroD factors are not able to target closed chromatin refuted.

While the high occupancy of NEUROD2 in dynamic chromatin regions, both *in vivo* and *in vitro*, along with the centrality of CAT-containing E-boxes in these regions, strongly suggests NEUROD2’s direct involvement in chromatin remodeling, it is important to note that NEUROD1, another bHLH factor with recognized pioneer capacity (109), and *Neurod6*, are largely co-expressed with *Neurod2* and could also potentially bind to the same E-boxes. This could explain why some centrally enriched E-boxes in transient chromatin regions *in vivo* do not show NEUROD2 binding in our dataset, even though we demonstrated the contribution of false negatives from NEUROD2 ChIP-seq datasets. The dissection of the individual contribution of each of these factors and their redundancy will require additional ChIP-seq datasets in primary tissue and more comprehensive experimental designs *in vitro*.

## Supplementary Material

gkae950_Supplemental_File

## Data Availability

All code required to perform the analyses of this study is deposited in GitHub (https://github.com/SantpereLab/neurog2-neurod2-neurogenesis) and Zenodo, the DOI is: 10.5281/zenodo.13898345.
