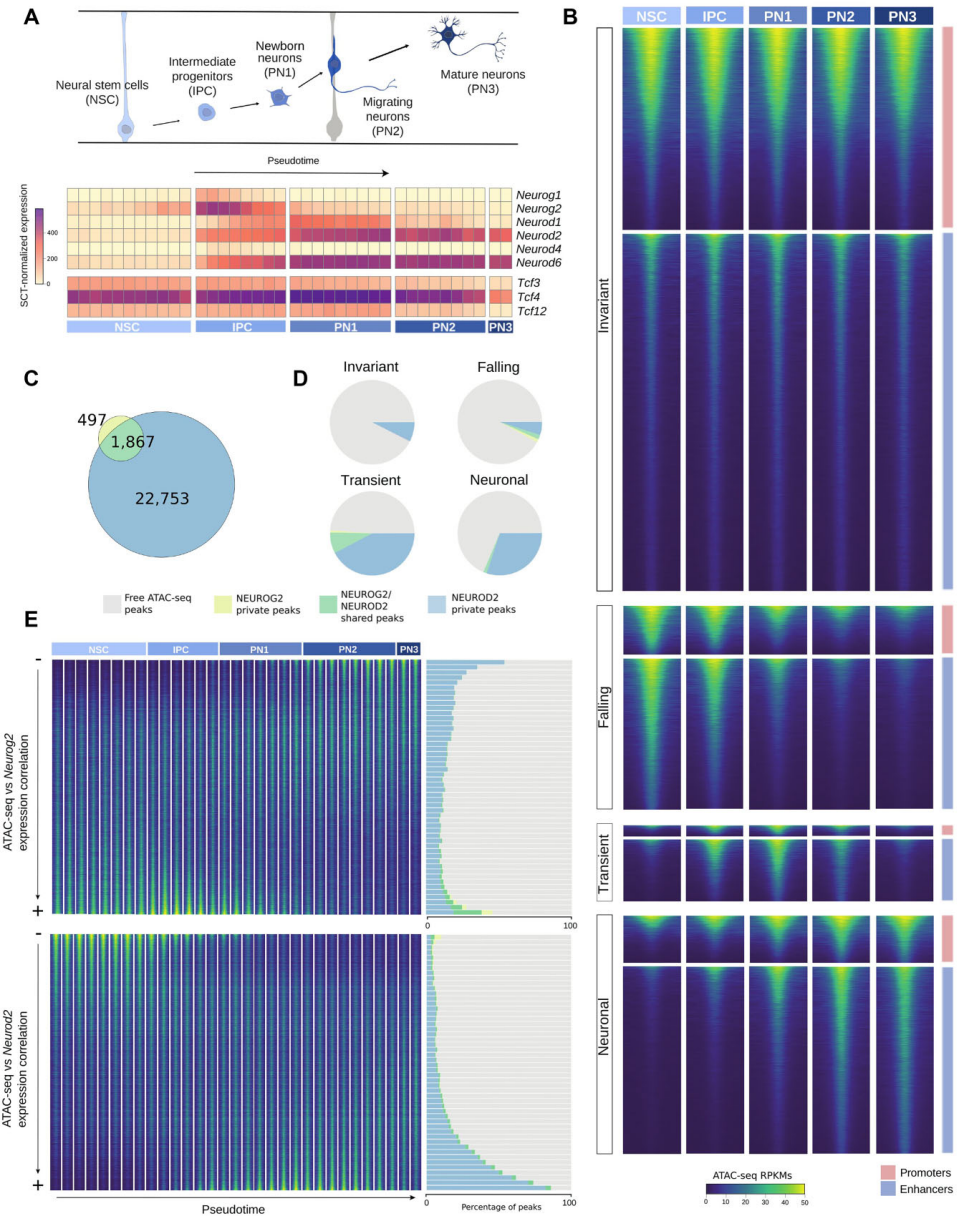# Correction to ‘Recruitment of homodimeric proneural factors by conserved CAT–CAT E-boxes drives major epigenetic reconfiguration in cortical neurogenesis’

**DOI:** 10.1093/nar/gkaf129

**Published:** 2025-02-18

This is a correction to: Xabier de Martin, Baldomero Oliva, Gabriel Santpere, Recruitment of homodimeric proneural factors by conserved CAT–CAT E-boxes drives major epigenetic reconfiguration in cortical neurogenesis, *Nucleic Acids Research*, Volume 52, Issue 21, 27 November 2024, Pages 12895–12917, https://doi.org/10.1093/nar/gkae950

In the originally published version of this manuscript, the authors inadvertently duplicated the barplot in Fig. 1 panel E during the proofreading process.

Additionally, the captions of Fig. 6 panels C and E have been updated from:

(C) Heatmaps representing the number of each type of E-box in regions within each decile of chromatin accessibility scaled across all deciles and E-boxes. Each row represents one of the induction studies included in B).

To

(C) Heatmaps representing the number of each type of E-box in regions within each decile of chromatin accessibility scaled across all deciles in each E-box. Each row represents one of the induction studies included in B).

And

(E) Heatmaps representing the number of each type of E-box in regions within each decile of pre- versus post-induction fold change of chromatin accessibility scaled across all deciles and E-boxes. Each row represents one of the induction studies included in D).

To

(E) Heatmaps representing the number of each type of E-box in regions within each decile of pre- versus post-induction fold change of chromatin accessibility scaled across all deciles in each E-box. Each row represents one of the induction studies included in D).

These changes do not affect the results, discussion and conclusions presented in the article. The published article has been updated.