# Supplementary material for: Recruitment of homodimeric proneural factors by conserved CAT–CAT E-boxes drives major epigenetic reconfiguration in cortical neurogenesis
Source: Nucleic Acids Res. 2024 Nov 4;52(21):12895–917. doi: 10.1093/nar/gkae950 (PMC11602148; doi:10.1093/nar/gkae950)
Supplement: gkae950_Supplemental_File [file gkae950_supplemental_file.pdf]

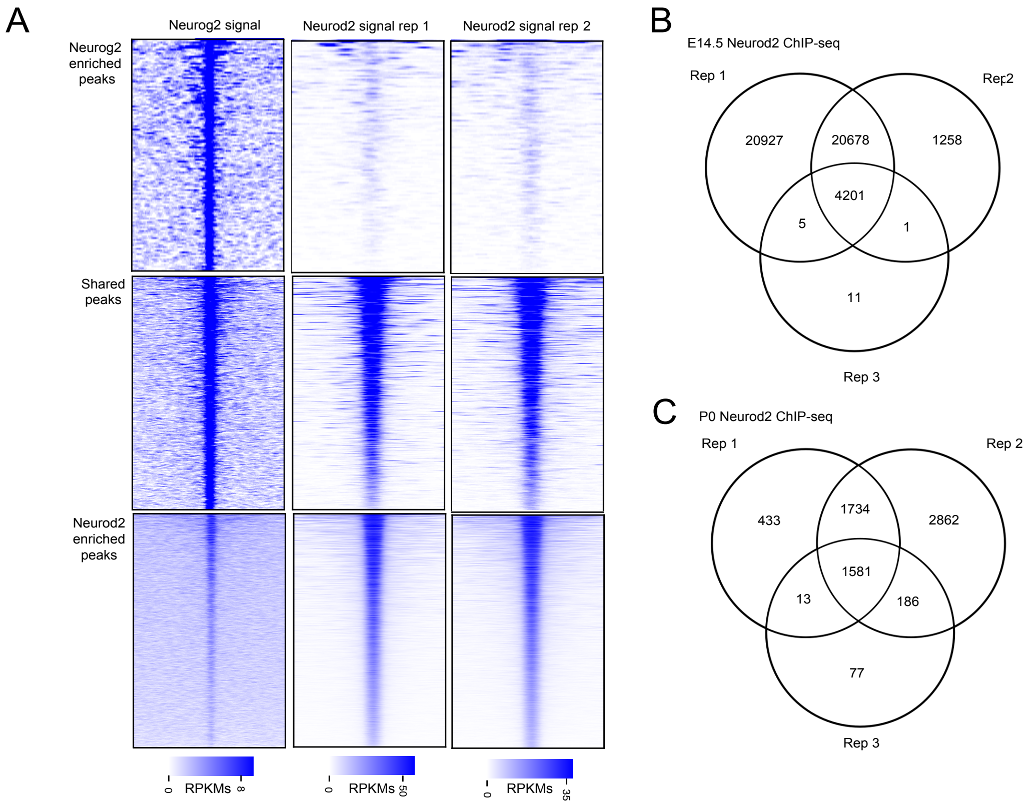

Supplementary Fig. 1. Comparison of ChIP-seq datasets used in this study. (**A**) ChIP-seq heatmaps depicting the signal of the binding of NEUROG2 and NEUROD2 in various categories of peaks (NEUROG2-enriched, NEUROD2-enriched or shared). (B-C) Venn diagram showing overlaps between ChIP-seq replicates performed in the NEUROD2 e14.5 and p0 mouse brain.

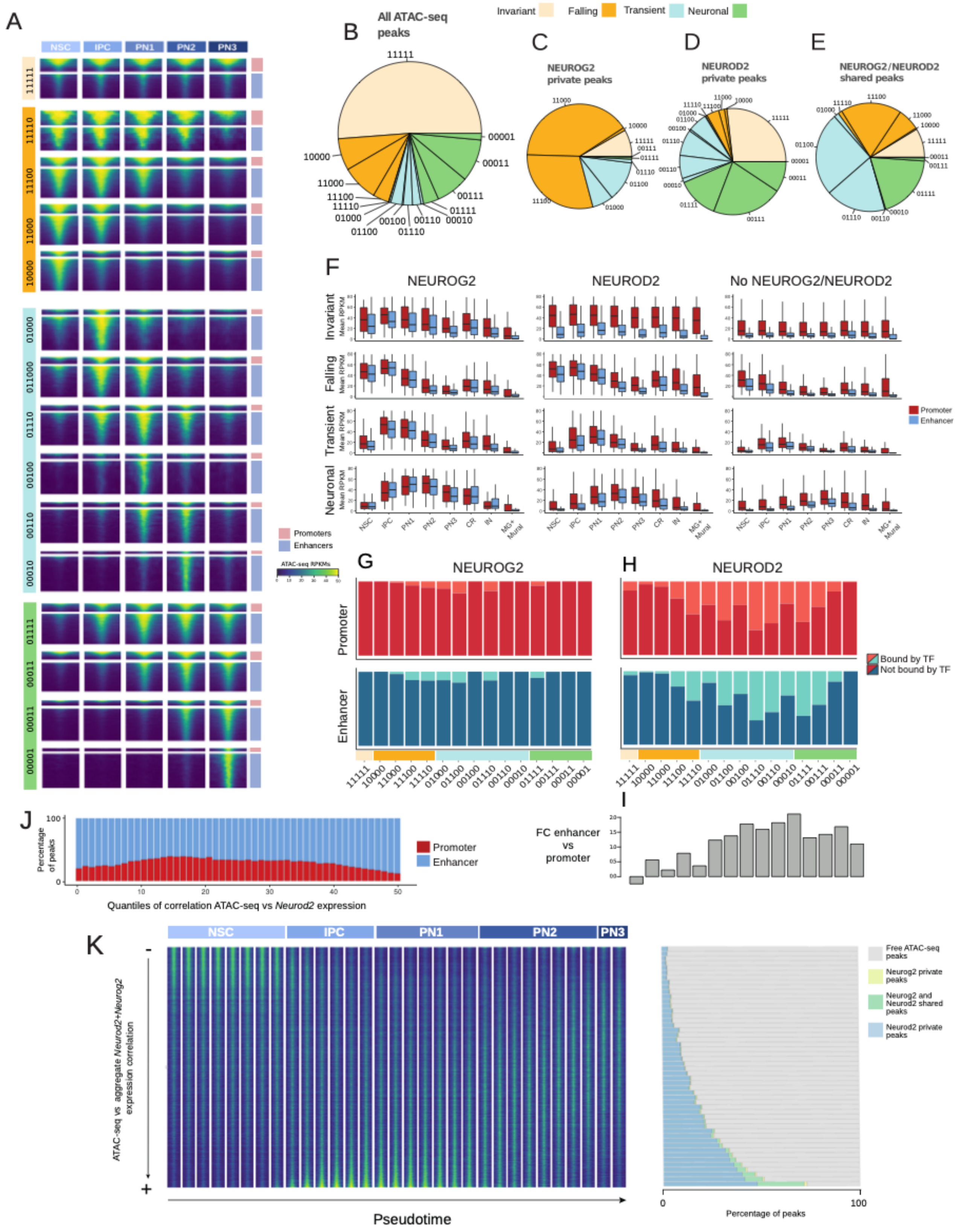

Supplementary Fig. 2. Detailed view on the binding of proneural factors in the landscape of chromatin accessibility of neurodevelopment. (**A**) Heatmaps depicting the ATAC signal in the different chromatin accessibility trajectories as directly derived from our correlation-based method (see methods) without the grouping in the major trajectories. (**B**) Pie chart showing the proportion of trajectories among all ATAC-seq peaks. (**C**) Pie chart of the proportion of trajectories among regions only bound by NEUROG2. (**D**) Pie chart of the proportion of trajectories among regions only bound by NEUROG2 and NEUROD2. (**E**) Pie chart of the

proportion of pseudotime trajectories among regions only bound by NEUROD2. (**F**) Boxplots showing the distribution of ATAC-seq signals in accessible chromatin peaks of each trajectory overlapping NEUROD2 peaks, NEUROG2 peaks, or none, in each cell type. (**G**) Barplots depicting the proportion of ATAC-seq peaks in each trajectory bound by NEUROG2, or (**H**) NEUROD2. (**I**) Barplot indicating the fold change of the proportion of NEUROD2-bound peaks in each trajectory in enhancers versus promoters. (**J**) Barplot representing the proportion of enhancers and promoters in ATAC-seq peaks grouped according to the correlation of their accessibility with the expression of *Neurod2*. (**K**) (Left) Heatmaps representing pseudobulk signal around the summits of the ATAC-seq peaks sorted by the correlation of their accessibility with the combined expression of *Neurog2*and *Neurod2*. (Right) Proportion of ATAC-seq peaks bound by NEUROG2/NEUROD2 private in each of 50 quantiles of the correlation with the corresponding transcription factor.

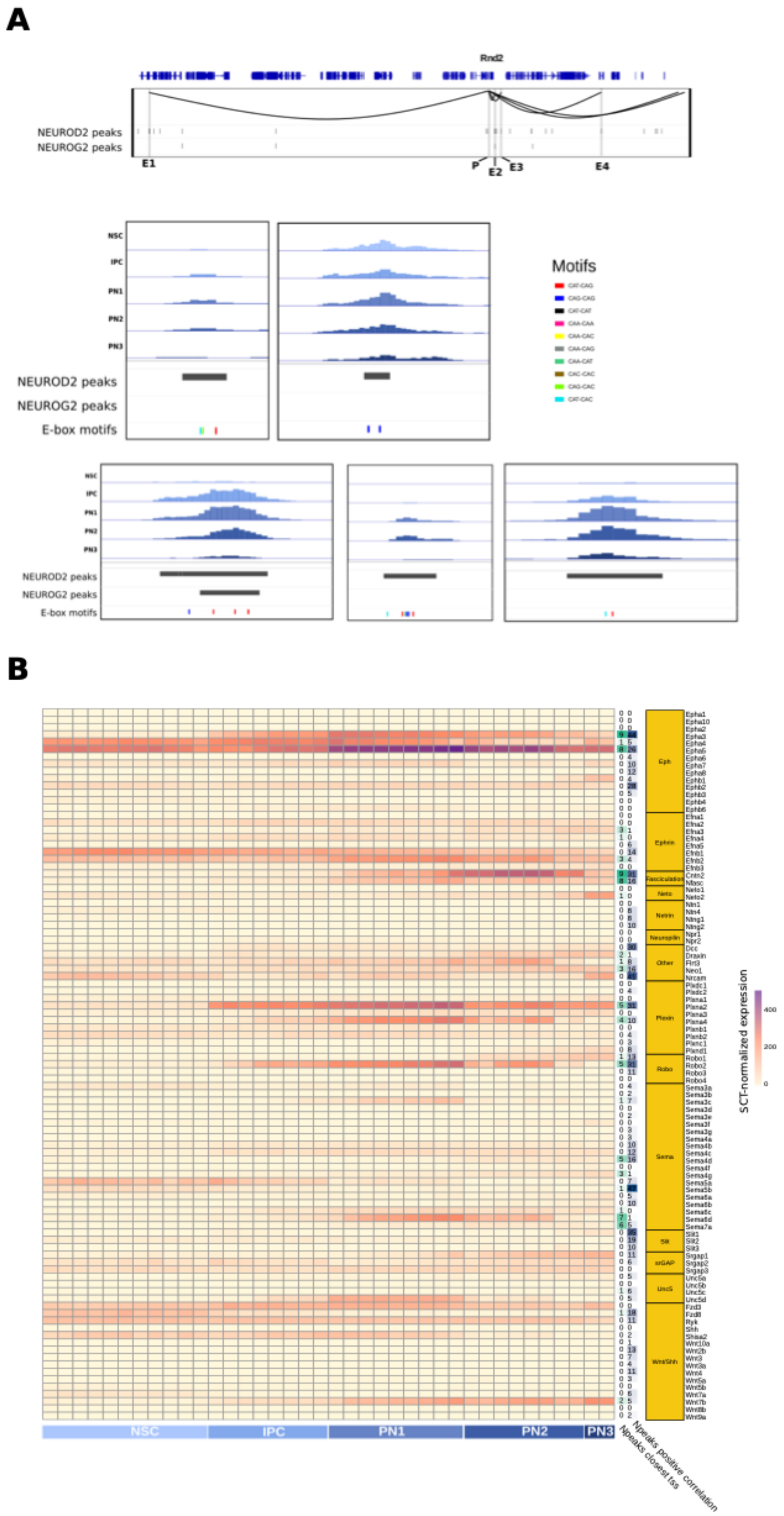

Supplementary Fig. 3 (**A**) On the top, the genomic region around the *Rnd2* gene is depicted, with the enhancers linked based on positive correlation with the expression. On the bottom, a closer view on the linked

enhancers that intersect with a NEUROD2 peak, showing the chromatin accessibility in neurodevelopment and the E-box motifs located in the peaks. (**B**) Heatmap representing average expression of axon guidance genes on pseudobulk samples ordered by pseudotime. Right columns contain the number of NEUROD2 peaks associated with each axon guidance gene (right), and the number of NEUROD2 peaks linked to the gene by accessibility-expression correlation (left).

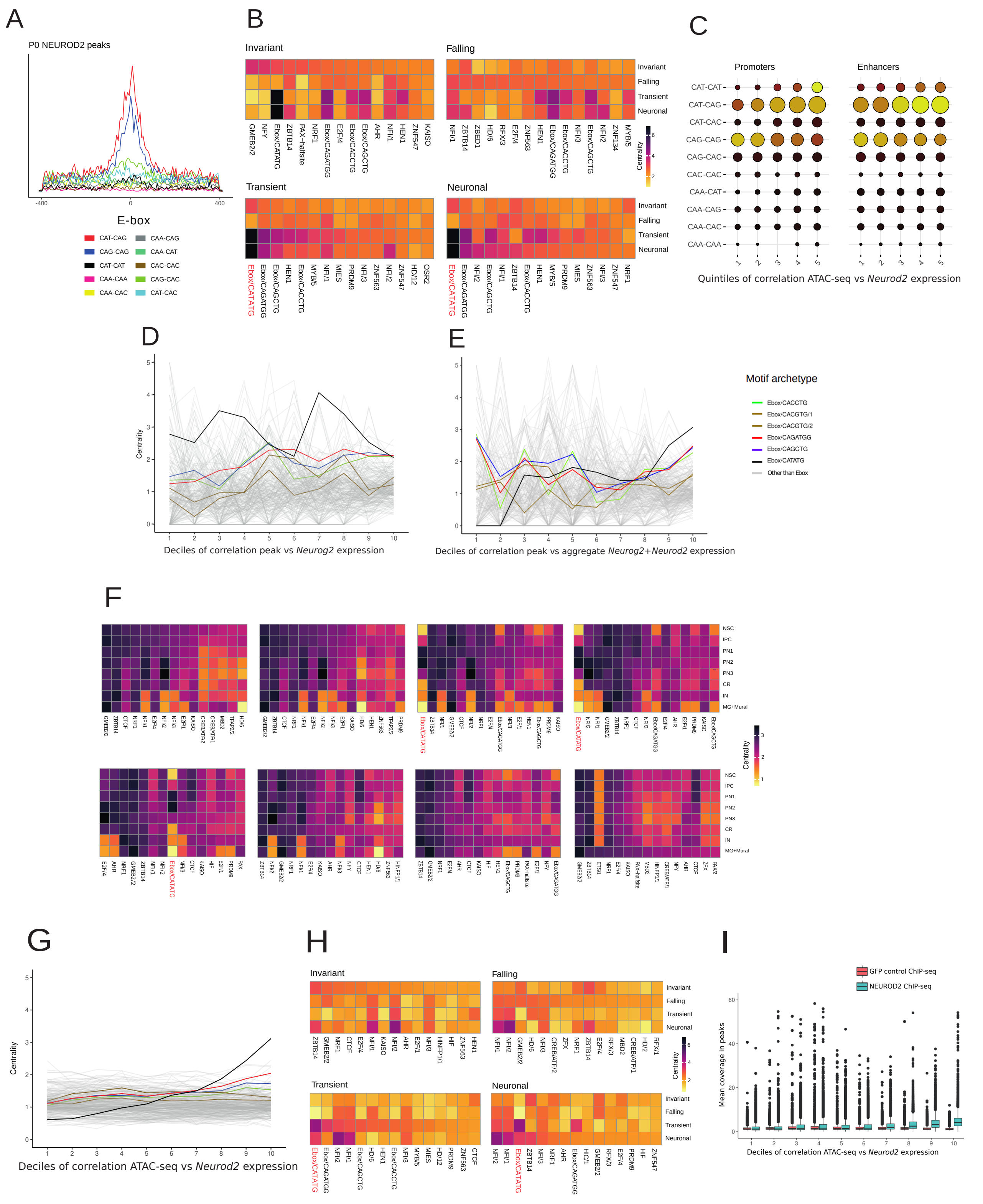

Supplementary Fig. 4 (**A**) Number of E-box motifs per base pair and per peak around the summits of NEUROD2 peaks in the P0 mouse cortex. (**B**) Heatmaps representing the centrality in the different trajectories of the top 15 most enriched motifs of each trajectory. (**C**) Centrality (motifs 50bp around the summit vs >150bp further) of each E-box type around the summits of the NEUROD2 peaks overlapping promoters and enhancers, binned by the correlation with *Neurod2* expression levels. (**D**) Line plot indicating motif centrality (motifs 50bp around the summit vs >250bp further) of all the archetypal motifs determined in Vierstra et al. around the

summits of the ATAC-seq peaks, grouped by the correlation of their ATAC accessibility with the expression levels of NEUROG2. (**E**) Line plot indicating motif centrality (motifs 50bp around the summit vs >250bp further) of all the archetypal motifs determined in Vierstra et al.(62) around the summits of the ATAC-seq peaks not bound by NEUROD2 and NEUROG2, grouped by the correlation of their ATAC accessibility with the aggregated expression levels of *Neurod2* and *Neurog2*. (**F**) Heatmaps showing the centrality of motifs around the summits of the peaks called independently in each cell type. In each heatmap, the top 15 most enriched motifs of each cell type are shown. (**G**) Line plot indicating motif centrality (motifs 50bp around the summit vs >250bp further) of all the archetypal motifs determined in Vierstra et al.(62) around the summits of the ATAC-seq peaks not bound by NEUROD2, grouped by the correlation of their ATAC accessibility with the expression levels of *Neurod2*. (**H**) The same as in (**B**) but looking at ATAC-seq peaks that do not intersect with NEUROD2 peaks. (**I**) Boxplot depicting NEUROD2 ChIP-seq signal in ATAC-peaks not bound by NEUROD2 as a function of the correlation of their ATAC accessibility with the expression levels of *Neurod2*, along with the signal of the GFP control of the ChIP-seq experiment for comparison.

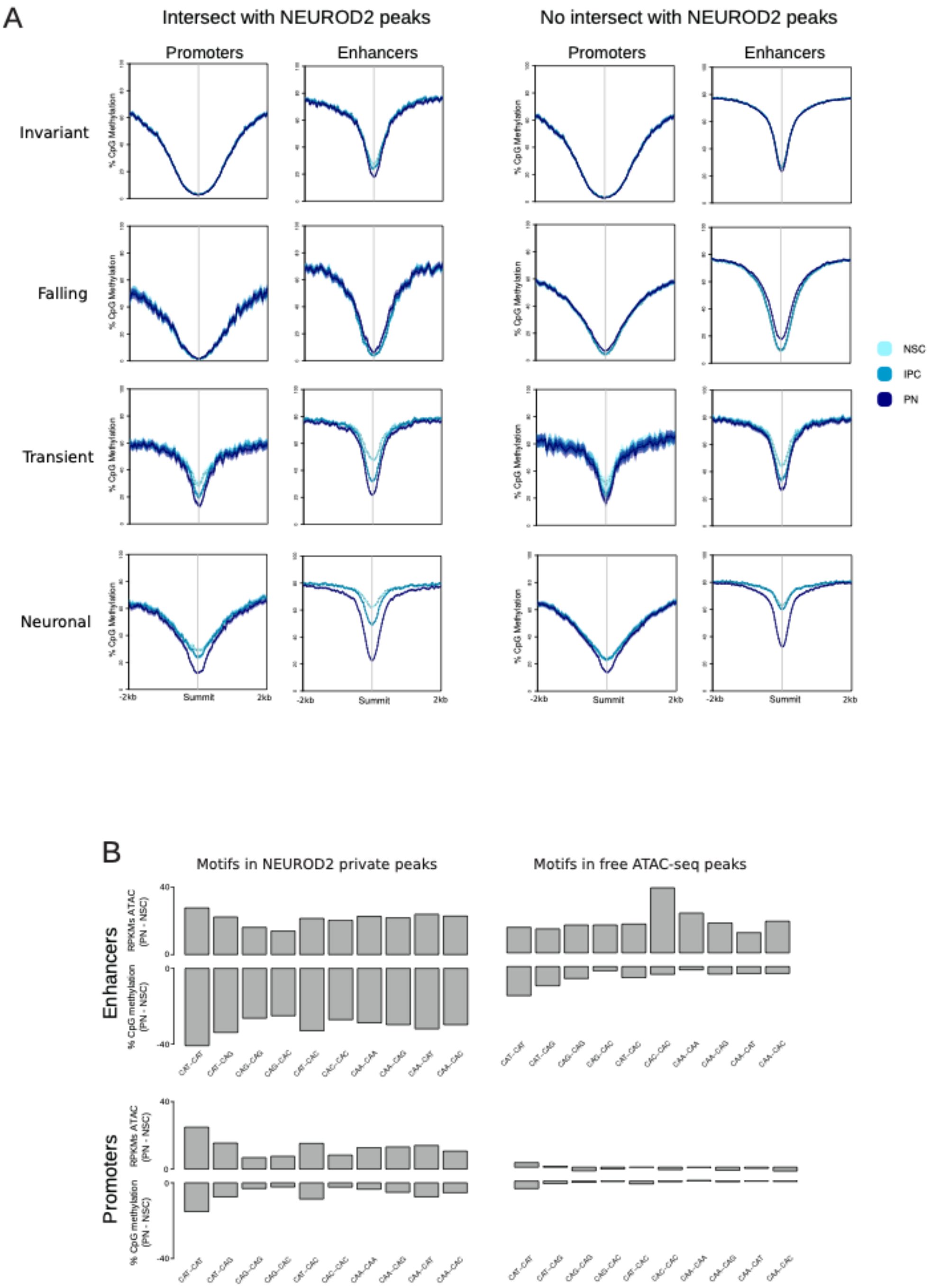

Supplementary Fig. 5 (**A**) Lineplot representing the average signal of the subsets of ATAC peaks in each trajectory bound or not bound by NEUROD2, with ribbons representing the standard error of the mean. (B) Barplots representing the difference in percentage of CpG methylation measured 5bp around each type of E-box between PN and NSC (bottom) in promoters and enhancers bound by NEUROD2. The top barplots represent the same comparisons using ATAC-seq RPKMs.

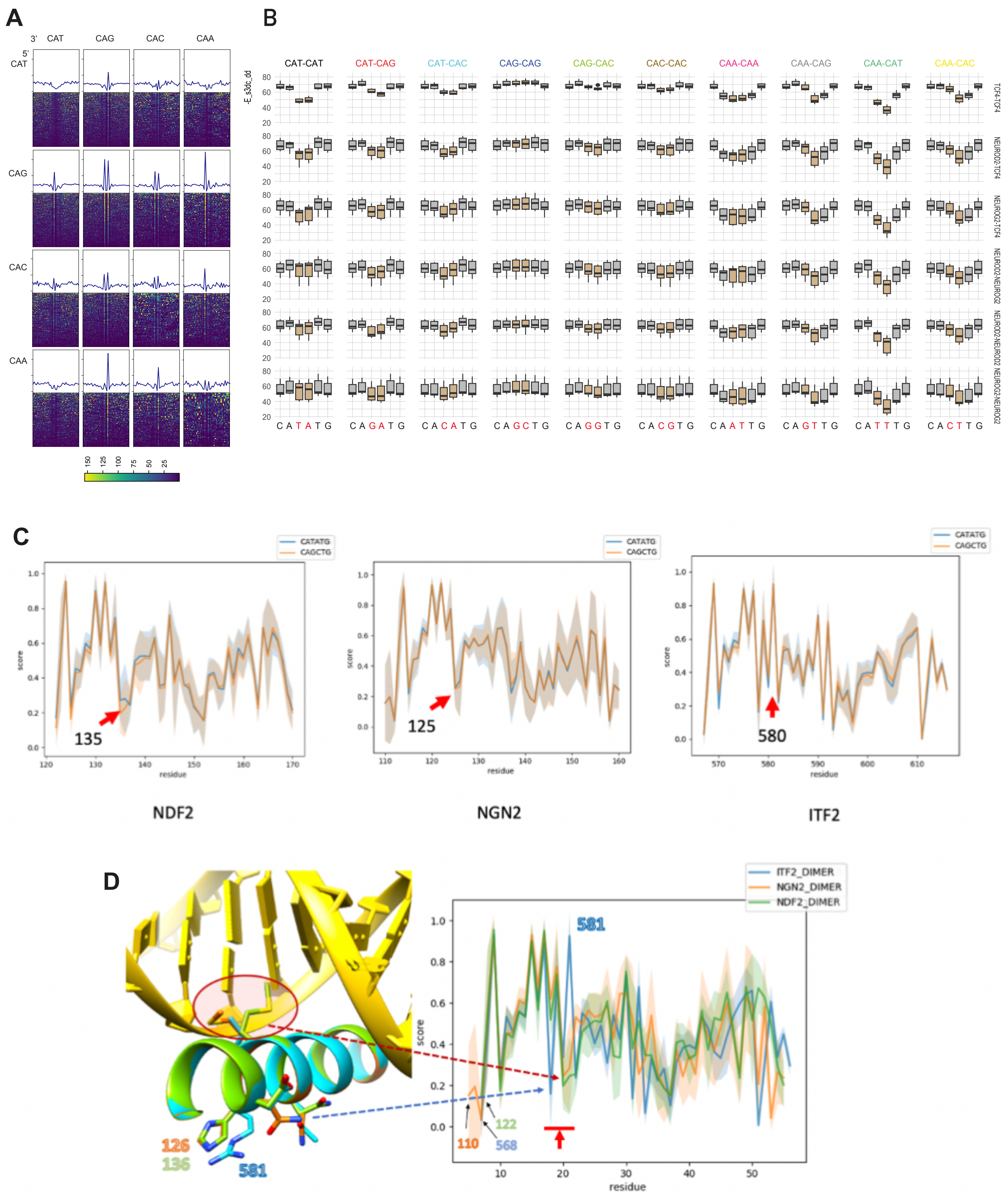

Supplementary Fig. 6 (**A**) Footprinting analysis showing HINT-normalized transposase cut frequency in aggregated (lineplot, up), or in individual peaks (heatmap, down), around all possible CANNTG hexanucleotides in regions bound by NEUROG2. (**B**) Boxpot showing the distribution of binding affinities at the nucleotide level for each dimeric composition and E-box, using multiple PDB structures as template. (C) The figures depict normalized interaction scores between DNA sites and amino acids of NEUROD2, NEUROG2, and TCF4 for CAT-CAT (blue) and CAG-CAG (orange) sequences. Red arrows highlight score differences and their position in the amino-acid sequence. (**D**) The plot at the right displays normalized scores per amino acid for NEUROD2 (green), NEUROG2 (orange), and TCF4 (blue) homodimers. On the left, a

ribbon-plot illustrates the modeled bound helix of NEUROD2 (green), NEUROG2 (orange), and TCF4 (blue), revealing encircled in red side-chains of Val-580 in TCF4 and Met-135/Met-125 in NEUROD2/NEUROG2. The visuals provide insights into sequence-specific interactions and structural distinctions among the analyzed transcription factors. Side-chains of Arg-581 in TCF4 and His-136/His-125 in NEUROD2/NEUROG2 provide reference points in the plot, with reassigned numbers for correct alignment.

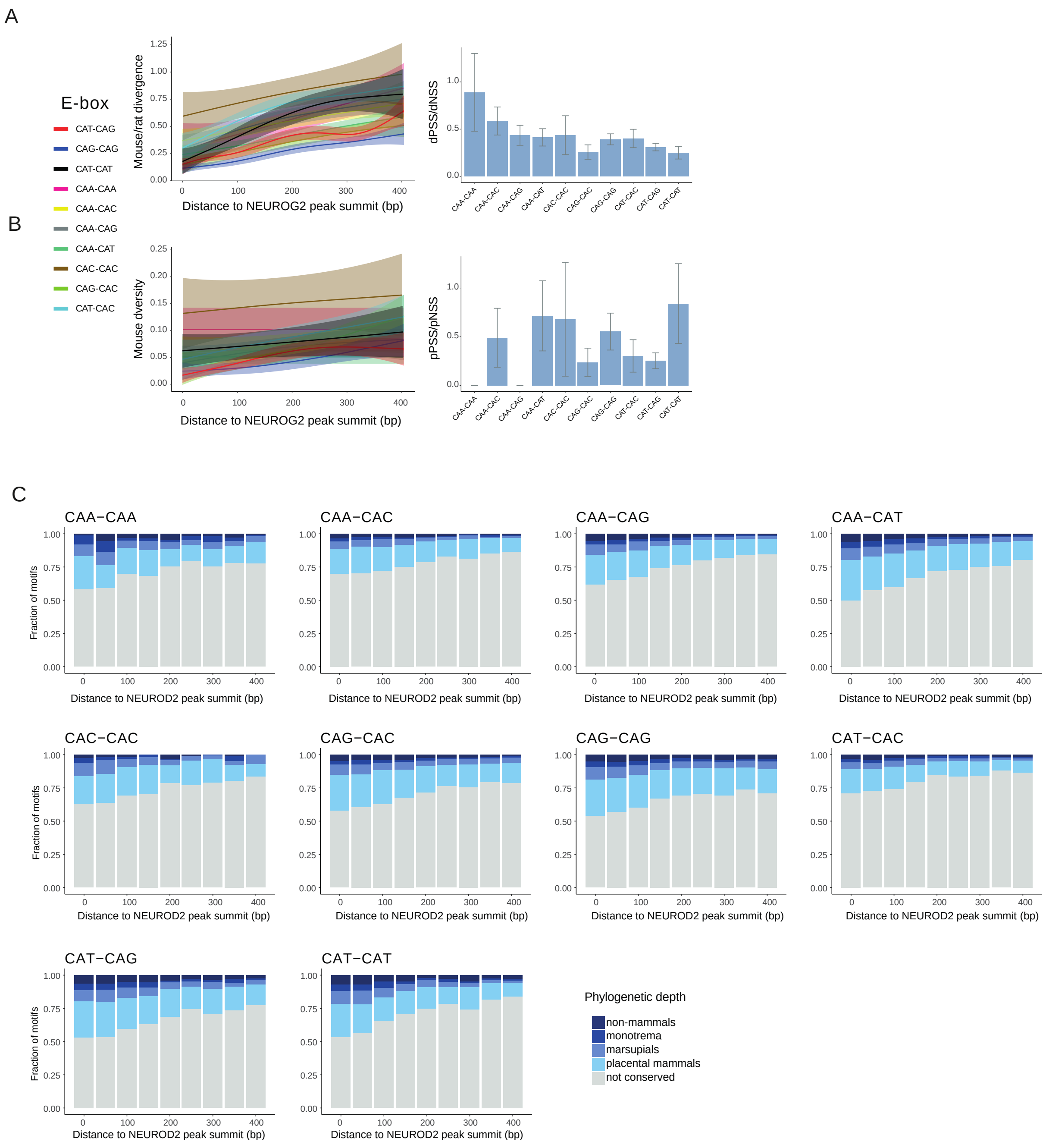

Supplementary Fig. 7 (**A**) Line plots displaying the number of mouse-rat substitutions within E-boxes in bins of increasing distance to NEUROG2 peak summits (left). Barplot showing the ratio of the dPSS and dNSS in each E-box type in NEUROD2 peaks (right). (**B**) Line plots showing number of SNPs within E-boxes in bins of increasing distance to NEUROG2 peak summit (left). Barplot showing the ratio of the pPSS and pNSS in each E-box type in NEUROD2 peaks (right). (**C**) Barplots representing the proportion of each type of E-boxes displaying various degrees of phylogenetic conservation at bins of increasing distance to NEUROD2 peak summits.

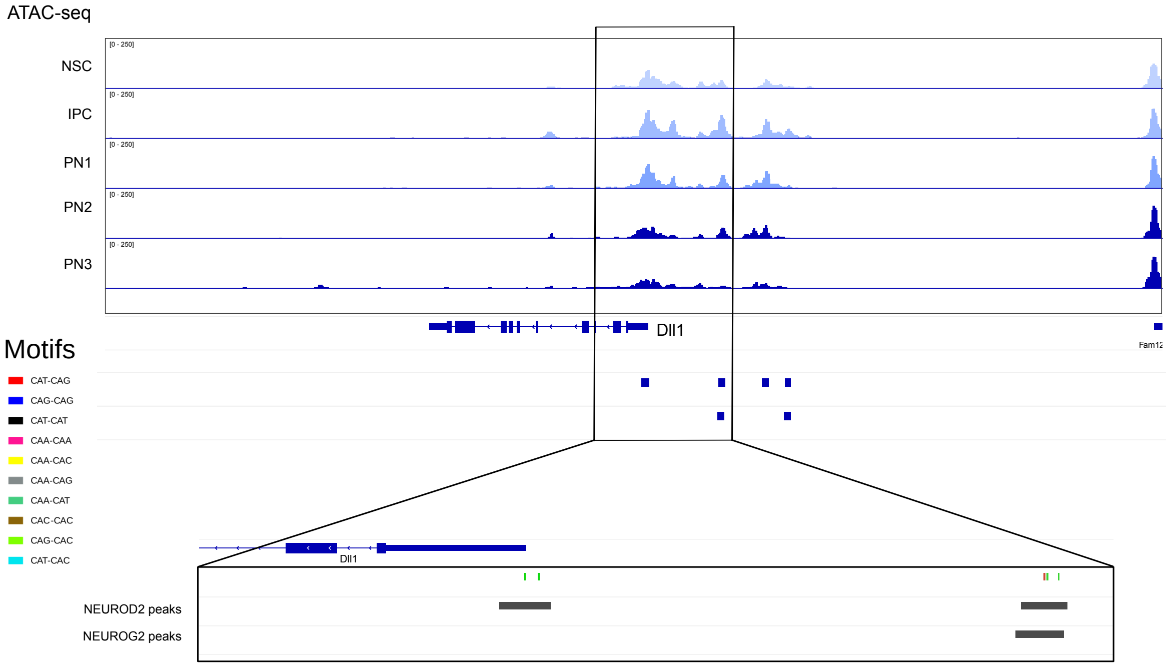

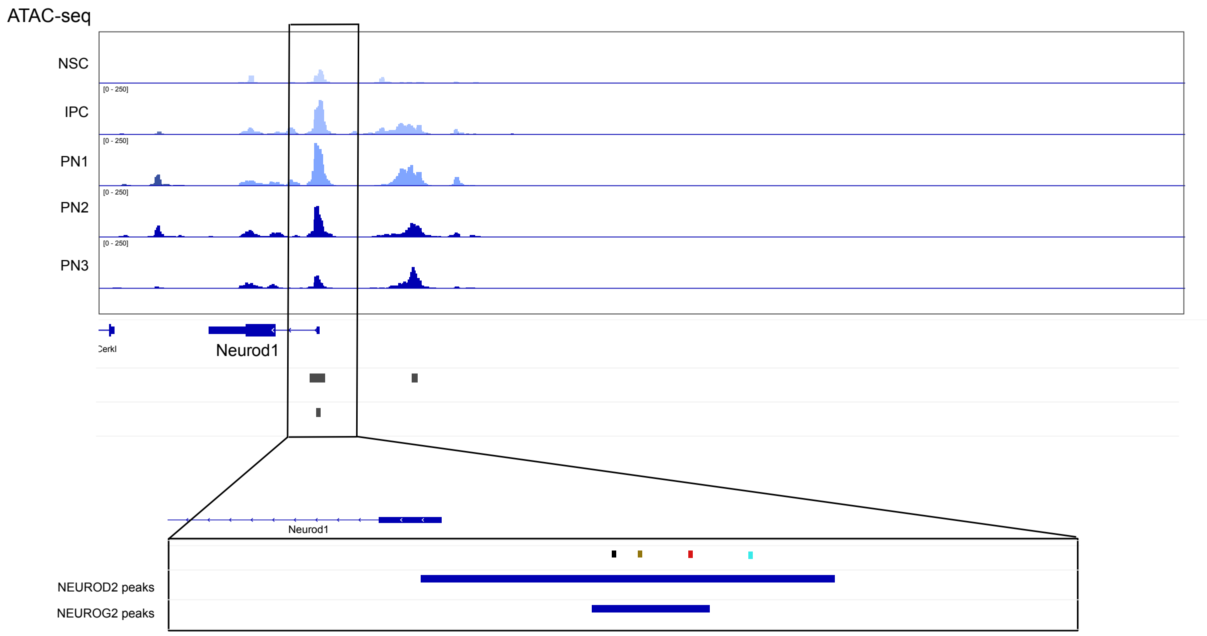

Supplementary figure 8. Genome browser representation of NEUROD2 and NEUROG2 peaks associated with the gene *Dll1* and *Neurod1* and their signal. Specific E-boxes overlapping highlighted peaks are represented.
